# Reducing concerns about falling in older adults: evaluation of a multi-component intervention and the role of stress and inflammation markers - long-term effects of the FEARFALL randomized controlled trial

**DOI:** 10.1007/s40520-026-03515-z

**Published:** 2026-09-19

**Authors:** Sabine Britting, Anja Müller, Robert Kob, Ellen Freiberger, Nicolas Rohleder

**Affiliations:** 1 Institute for Biomedicine of Aging (IBA), Department of Internal Medicine-Geriatrics, Friedrich-Alexander-Universität Erlangen-Nürnberg, Nuremberg, Germany; 2 Chair of Health Psychology, Department of Psychology, Friedrich-Alexander- Universität Erlangen-Nürnberg, Erlangen, Germany

**Keywords:** Older adults, Concerns about falling, Inflammation, Community-dwelling, Stress, Cortisol

## Abstract

**Background:**

Concerns about falling (CaF) are common in older adults and associated with reduced activity and poorer health. This study investigated, for the first time, the long-term effects of a multi-component intervention targeting CaF on psychological, functional, biophysiological, and anthropometric outcomes.

**Methods:**

160 community-dwelling older adults (≥ 70 years) were randomly assigned to a 4-month intervention featuring exercise and cognitive-behavioral training or to a sham control group. CaF and related psychological outcomes were assessed using validated questionnaires. Salivary cortisol and α-amylase, physical function, activity, body composition and inflammatory markers (C-reactive protein [CRP]-, interleukin-6 [IL-6], interleukin-10 [IL-10], interleukin-1β [IL-1β] and tumor necrosis factor-alpha [TNF-α]) were analyzed at baseline, post-intervention, and 8-month follow-up.

**Results:**

All estimates represent T0–T2 estimated marginal mean differences. There was no intervention effect or long-term superiority between the groups. Significant time effects were observed for CaF and physiological biomarkers for stress axis activity and inflammatory markers. Functional outcomes changed only marginally.

**Conclusion:**

CaF improved similarly in both groups. Changes in basal stress system activity and inflammatory cytokines were highly comparable between groups. Contrary to our hypothesis, the intervention did not confer additional benefits over the sham control intervention in reducing CaF.

**Trial registration:**

German Clinical Trials Register (DRKS): DRKS00029171. Registered 22 July 2022, https://drks.de/search/de/trial/DRKS00029171.

**Supplementary Information:**

The online version contains supplementary material available at https://doi.org/10.1007/s40520-026-03515-z.

## Introduction

Healthy aging and mobility play a crucial role in enabling older adults to preserve their independence. This significance is further emphasized by the World Health Organization´s (WHO) proclamation of the Decade of Healthy Aging 2021 to 2030 [1], defining healthy aging as “the process of developing and maintaining the functional ability that enables well-being in older age”. The framework by the WHO on healthy aging includes the association between intrinsic capacity and functional ability taking environmental factors and public health systems into account. Therefore, optimizing older people’s functional ability is a key factor in reaching the WHO´s goal of healthy aging during this decade.

Falls are a major public health issue threatening healthy aging and independence in older adults, with a global fall prevalence of 26.5% [2], frequently leading to substantial physical impairment, increased morbidity and even sometimes death [3]. Falls are associated with concerns about falling (CaF) [4], widespread globally with a prevalence of 49.6% as shown in a systematic review and meta-analysis by Xiong et al. (2024) [5]. CaF negatively affect physical activity and mental well-being contributing to a downward spiral of future falls, reduced activity, declining muscle mass and function (sarcopenia) and further increases CaF [6] as well as decreased quality of life [7], depression [8] and social isolation [9].

CaF exhibit notable similarities to psychological conditions like anxiety and may lead to physiological effects akin to those caused by chronic stress [10]. However, direct evidence that CaF per se causes sustained dysregulation of the hypothalamic-pituitary-adrenal (HPA) axis or sympathetic nervous system (SNS), or measurable shifts in systemic inflammatory markers, remains limited. Chronic stress is characterized by dysregulations in physiological stress systems, such as reduced (HPA) axis activity and increased sympathetic nervous system (SNS) activity, which further promote inflammatory activity and persistent low-grade inflammation [11–14]. Inflammation is a fundamental component in the pathophysiological processes of age-related diseases such as frailty and sarcopenia [15].

Research on sarcopenia has highlighted the detrimental impact of inflammatory processes on muscle quality and function, further contributing to the negative spiral of deterioration of health [16]. Chronic low-grade inflammation, in the context of aging also referred to as “inflammaging” [17], is a crucial factor in the progression of sarcopenia, characterized by age-related loss of muscle mass and function. Elevated inflammatory molecules such as interleukin 6 (IL-6), tumor necrosis factor-alpha (TNF-α) and C-reactive protein (CRP) are known to accelerate muscle protein breakdown, inhibit muscle synthesis through mammalian target of rapamycin (mTOR) pathways, and impair muscle regeneration [18, 19].

Despite a robust understanding of these physiological mechanisms, less is known about interventions addressing psychological dimensions of falls. While various studies have explored the immediate impact of interventions on reducing CaF [20, 21], there is a notable gap in research regarding the long-term effects of these interventions on CaF, biomarkers and physical function.

The FEARFALL study (“Chronic stress and functional health in older adults with concerns about falling”) investigates how fall-related psychological concerns—conceptualized as a chronic stressor—are associated with functional health in older adults. Here we examined whether changes in CaF, the basal stress system activity as well as inflammatory markers are related over a total observation period of 12 months (baseline, post-intervention and 8-month follow-up). This research is conducted within the framework of a multi-component intervention that includes exercise training and cognitive-behavioral components targeting CaF in community-dwelling older adults. Building on the short-term effects previously reported by Müller et al. (2025) [22], this study aims to present the long-term effects of the intervention. It is hypothesized that reductions in CaF and related psychological symptoms including perceived stress, depression, quality of life and anxiety, achieved during the intervention, will remain stable at the 8-month follow-up, and will be accompanied by a normalization of basal stress axis activity and reductions in low-grade inflammation.

## Methods

### Study design

This study reports the longitudinal data from a randomized controlled trial examining the relationship between chronic stress and functional health in older adults with concerns about falling (FEARFALL study). The FEARFALL study was registered in the German Clinical Trials Register (DRKS) under registration number DRKS00029171 on 22 July 2022 (https://drks.de/search/de/trial/DRKS00029171). The study protocol was published by Britting et al. (2024) [23] and was pre-registered on the Open Science Framework (OSF) https://doi.org/10.17605/OSF.IO/R4CDX). The ethics committee of Friedrich-Alexander-Universität Erlangen-Nürnberg granted approval for the study on August 26, 2020 (protocol 317_20 B), and written informed consent was provided by all participants. All study procedures adhered to the Declaration of Helsinki. The CONSORT reporting guidelines and checklists are included in Supplementary Material 1 (SM 1) [24].

### Participants

In this study, community-dwelling older adults aged 70 and older were invited to take part in a four-month intervention program. Interested individuals were initially screened based on predetermined inclusion criteria, including being 70 years or older, scoring ≥ 20 on the Falls Efficacy Scale-International (FES-I) [25, 26], the ability to attend the study center independently, consent to randomization, and providing informed consent. Exclusion criteria included having a life expectancy of less than 12 months, being concurrently enrolled in another intervention study (physical or medical exercise programs) within the past six months, using anti-inflammatory medications (such as glucocorticoids or NSAIDs), or having cognitive impairment (Mini Mental State Examination (MMSE) < 24 points).

### Measures

A detailed description of the specific instruments employed in the FEARFALL study is available in the study protocol [23] and the preregistration (https://doi.org/10.17605/OSF.IO/R4CDX). With the exception of demographic variables, all measurements were collected at three time points, at baseline (T0), after the intervention (T1) and at 8-month follow-up (T2). A flow chart of study population is provided as Supplementary Material 2 (SM 2).

#### Demographic variables

At baseline assessment, demographic information, age, sex and current living circumstances were assessed. At each assessment, fall events since the last measurement were recorded: at baseline, falls during the previous 12 months; at T1, the preceding 4 months; and at T2, the preceding 8 months. To ensure comparability across assessment time points, number of falls was standardized to a 12-month period.

#### Psychological variables

To gain a comprehensive understanding of various dimensions of falling concerns, we operationalized the construct across three domains: concerns about falling, activity avoidance and perceived control aspects [26–28].

CaF were assessed by the Falls Efficacy Scale-International (FES-I) [26]. Behavioral consequences of CaF were investigated by the currently validated FES-IAB, asking for the avoidance behavior regarding physical activity for all FES-I items [27]. Perceived control over falling was assessed by the Updated Perceived Control over Falling (UP-CoF) [28].

To characterize participants’ mental health, we measured anxiety using the German version of Geriatric Anxiety Scale (GAS-G) [29], depression using the Geriatric Depression Scale (GDS-15) [30] and stress using the Perceived Stress Scale 10 (PSS) [31], and health related quality of life using EURQOL Visual-Analogue-Scale (EQ-5D VAS) [32].

#### Functional measurements

Handgrip strength was used as a surrogate parameter for muscle strength assessed with a Jamar hydraulic hand dynamometer (Sammons Preston Rolyan, Bolingbrook, IL, USA) according to the EWGSOP2 recommendations [33].

The Short Physical Performance Battery (SPPB) was used to assess physical performance across three lower-limb functional tasks: gait speed (m/s), chair rise (time to complete five sit-to-stand repetitions in seconds), and static balance (time in seconds held across progressively challenging positions) [34].

Physical activity (PA) was recorded by a seven-point scale for assessing self-rated physical activity especially for older adults [35, 36].

The activPAL system (PAL technologies, Glasgow, UK) was used to objectively assess physical activity through actigraphy. Data were included in the analysis if at least four complete days (without non-wear time) were recorded using the CREA v1.3 algorithm.

#### Stress system activity and inflammation

Diurnal profiles of salivary cortisol and alpha amylase (sAA) were assessed following established protocols to assess basal HPA axis and SNS activity [13]. At the three time points (T0, T1 and T2) participants collected saliva samples at home on two consecutive days (D1; D2) at wake-up (S1), 30 min after waking (S2), and bedtime (S3) using Salivette collection devices (Sarstedt, Nümbrecht, Germany). All samples with a deviation of more than five minutes (S2) or sampling ahead of the prescribed time of S3 (< 14 h) were excluded. Cortisol concentrations were determined in duplicate according to manufacturers´ instructions using a chemiluminescence immunoassay (CLIA, IBL International GmbH, Hamburg, Germany). Salivary alpha-amylase was measured using an in-house enzyme kinetic assay as described in more detail in previous publications [37]. Basal stress system activity was quantified using three indices, based on the mean of corresponding samples from both days: the awakening response of cortisol and alpha-amylase (CAR/AAR), defined as the concentration change within 30 min after waking up [38, 39], the diurnal slope and the area under the curve with respect to ground (AUCg), representing total diurnal output [39].

In order to assess low-grade inflammation, blood samples were taken by the study physician using EDTA vacutainers (S-Monovette, Sarstedt) and an indwelling venous catheter (Safety-Multifly-Kanüle, Sarstedt). Quantification of IL-6 and CRP was performed using high-sensitivity ELISA kits (Quantikine HS, R&D Systems, Minneapolis, MN, USA; CRP High Sensitive ELISA, IBL International GmbH, Hamburg, Germany) according to the manufacturers’ instructions. The lower limits of detection were 0.09 pg/mL for IL-6 and 0.044 µg/mL for CRP. The intra-assay and inter-assay coefficients of variation (CV) were consistently less than 10%.

The inflammatory cytokine IL-1β and TNF-α as well as the anti-inflammatory cytokine IL-10 were measured on the Meso Scale Discovery^®^ S-Plex Proinflammatory Panel 1 (human) Kit, MESO QuickPlex SQ 120MM, Meso Scale Discovery^®^ (MSD) electrochemiluminescence multi-spot assay platform (MesoScale Diagnostics). Plasma samples were measured in duplicate using 25 µL of undiluted plasma per replicate according to the manufacturers’ instructions. The lower limits of detection were 71 fg/mL for IL-1β, 11 fg/mL for TNF-α and 19 fg/mL for IL-10. The intra-assay and inter-assay coefficients of variation (CV) were less than 10%.

#### Anthropometrics

Muscle mass was measured with bioelectrical impedance analysis (BIA; Seca Medical Body Composition Analyzer 515, Seca, Hamburg, Germany). Skeletal muscle mass (SM) was estimated using Janssen’s equation:

$$\begin{aligned}{\text{SM }}\left( {{\mathrm{kg}}} \right){\text{ }} =& {\text{ }}\left[ {\left( {{\mathrm{Ht}}^{2} /{\mathrm{R}}} \right){\text{ }} \times {\text{ }}0.{\mathrm{4}}0{\mathrm{1}}} \right]{\text{ }} + {\text{ }}\left( {{\text{gender }} \times {\text{ 3}}.{\mathrm{825}}} \right){\text{ }} +\\& {\text{ }}\left( {{\text{age }} \times {\text{ }} - 0.0{\mathrm{71}}} \right)\, + \,{\mathrm{5}}.{\mathrm{1}}0{\mathrm{2}}\end{aligned}$$

where Ht is height in centimeters; R is BIA resistance in ohms; for gender, men = 1 and women = 0; and age is in years. The skeletal muscle index (SMI) normalizes SM to body height [40].

### Multicomponent intervention

#### Intervention group (IG)

Participants assigned to the intervention group (IG) completed a 16-week, multicomponent exercise program; detailed procedures are described in the published study protocol [23]. Adherence was deemed satisfactory if participants attended at least 75% of scheduled sessions. The program targeted physical function through progressive strength, balance, gait, and functional task training, and incorporated cognitive-behavioral strategies adapted from the “A Matter of Balance” program [41]. The CaF component addressed goal setting, reduction of avoidance behaviors, and enhancement of self-efficacy.

#### Sham control group (SCG)

The SCG adhered to the same 16-week schedule. Each session comprised a 40-minute health-education lecture followed by 20 min of light upper-body stretching.

#### Randomization

Randomization was implemented using the Sealed Envelope online tool (Sealed Envelope Ltd., London, UK) by an independent member of the intervention team. To support blinding of outcome assessors and the statistician, intervention delivery and outcome evaluation were conducted by separate teams. Participants were informed that two interventions were being compared. Nevertheless, the possibility that participants could identify their group assignment cannot be ruled out. Allocation was stratified by gender and statin use to ensure balanced groups, and couples were block-randomized to enable joint participation.

#### Statistics

All statistical analyses were performed using R version 4.2.2 using the *lme4*, *lmerTest*, *emmeans*, *dplyr*,* tidyr and tidyverse* packages for model estimation, and visualization was performed using *ggplot2* and *sjPlot*.

Longitudinal data with three measurement points (T0, T1, T2) in two groups (IG vs. SCG) were analyzed. This study presents the results of the per-protocol analysis of those persons with a successful attendance. An intention-to-treat (ITT) analysis was also performed. The complete ITT results are provided in Supplementary Material 4 (SM 4). Age (z-standardized) and sex were included as covariates. For biomarker measurements, blood and saliva samples were collected at the scheduled assessment points. If sampling was not possible at the respective time point, samples were collected subsequently whenever feasible, typically within a maximum of two weeks. Remaining unavailable values were treated as missing and not imputed. Because of outcome-specific missingness, an (RM-) MANOVA/MANCOVA approach would have required complete-case analyses, resulting in considerable data loss and non-comparable analysis samples across outcomes. Therefore, primary inference was conducted on an outcome-by-outcome basis using linear mixed-effects models, which include all available observations per outcome.

$$\:Y\sim\:\mathrm{Group}\times\:\mathrm{Time}+{\mathrm{Age}}_{z}+\mathrm{Sex}+(1\mid\:\mathrm{SubID})$$

Time was modelled as a categorical factor (T0, T1, T2). Models were estimated using maximum likelihood (REML = FALSE), effects of Time, Group, and the Time × Group interaction were evaluated using Type III F-tests (*lmerTest*, sum-to-zero contrasts) with Satterthwaite-approximated degrees of freedom. More complex random-slope specifications proved unstable or non-identifiable due to the unbalanced design and outcome-specific missingness. Therefore, a random-intercept structure (1∣SubID) was used throughout. For one outcome, a singular (“boundary”) fit occurred, indicating a near-zero random-intercept variance. Model assumptions were evaluated by inspection of residual distributions and residual variance. No relevant violations of model assumptions were identified. Biomarker variables were transformed prior to analysis to reduce skewness and improve distributional properties: biomarker variables were log-transformed, except amylase variables, which were square-root transformed.

To control for multiple testing, p-values were adjusted separately for each effect (Time, Group, Time × Group) within four a priori defined content domains (psychological, functional, biomarker, and anthropometric outcomes) using the Benjamini–Hochberg procedure (false discovery rate, FDR). Domain-level results are presented for interpretive purposes only and do not replace outcome-specific inference. In total, 31 outcomes were analyzed, resulting in 93 outcome-level omnibus tests. Thus, FDR rather than the family-wise error rate was controlled. Post hoc contrasts were computed and were Benjamini–Hochberg adjusted within each contrast family. To quantify the magnitude of the observed effects, semi-partial R² values were calculated for the fixed effects Group, Time, and Time × Group and are reported together with the FDR-adjusted p-values in Supplementary Material 3 (SM 3). In addition, for concise domain-level summaries, an aggregated domain p-value per effect was computed from the outcome-specific p-values using the Aggregated Cauchy Association Test (ACAT), which combines multiple p-values into a single domain-level p-value while allowing for dependence among tests. To provide a concise and space-saving presentation in the main manuscript, only outcomes reaching FDR significance, and only the corresponding post hoc contrasts for those significant outcomes, were reported. Complete results for all analyzed outcomes are provided in the Supplementary Material 3 (SM 3).

## Results

Table 1 displays the baseline characteristics of participants by group, the IG (*n* = 87) and the SCG (*n* = 73), with no significant differences between the groups at baseline On average, the groups were similar in age and gender distribution. A high attendance rate (≥ 75%) was achieved in both groups.

**Table 1:** Characteristics of the study sample

	IG (*n* = 87)		SCG (*n* = 73)		
Sociodemographics					
	*n*	*%*	*n*	*%*	*p*
Women (n/%)	64	73.60	54	74.00	0.519
Living alone (n/%)	51	58.60	37	50.70	0.341
Falls (n/%)	58	66.70	44	60.30	0.414
Injurious falls (n/%)	36	41.40	31	42.50	0.890
Successful attendance ≥ 75% (n/%)	67	77.00	60	82.20	0.441
	*mean*	*SD*	*mean*	*SD*	*p*
Age [years]	79.83	5.59	79.16	4.92	0.431
Medications ^**a**^ [prescribed]	4.34	3.56	4.14	2.75	0.696

Anthropometrics					
BMI [kg/m^2^]	26.31	4.47	26.91	3.97	0.386
Skeletal Muscle Mass [kg]	21.65	3.40	21.45	3.14	0.704
Skeletal Muscle Index (kg/m^2^)	7.60	0.70	7.63	0.88	0.762

Psychological measurements					
FES-I [score long form, 16–64 p]	30.36	8.29	29.79	7.81	0.662
FES-IAB [score long form, 16–64 p]	25.03	7.45	24.77	8.01	0.827
UP-COF ^#^ [score, 0–20 p]	13.77	4.32	13.1	3.97	0.345
GAS [score, 0–75 p]	13.69	7.44	15.32	8.30	0.194
GDS [score, 0–15 p]	1.98	1.59	2.58	2.68	0.082
PSS [score, 0–40 p]	14.94	6.88	14.63	6.80	0.774
EQ-5D VAS [scale, 0-100]	62.63	17.54	64.3	15.89	0.532

Functional measurements					
handgrip strength [kg]	24.58	8.63	23.82	8.01	0.580
SPPB [score, 0–12 p]	8.85	2.63	8.75	2.45	0.805
SPPB 4-m gait speed [m/s]	0.87	0.19	0.84	0.23	0.392
SPPB chair rise time [seconds]	11.31	5.81	12.28	6.12	0.304
SPPB-Balance [score, 1–4 p]	3.33	1.74	3.15	1.51	0.490
PA-Level [1–7]	1.90	0.65	1.83	0.38	0.559
Step count	7227.49	3224.36	7207.36	3308.55	0.970

Biomarkers (saliva/blood) *					
CAR	0.10	0.23	0.10	0.17	0.957
Cortisol Slope	-0.03	0.15	-0.03	0.13	0.973
Cortisol AUCg	11.08	2.47	10.96	1.97	0.753
AAR	-1.69	2.69	-1.30	2.34	0.404
Amylase Slope	0.05	0.20	-0.03	0.12	0.807
Amylase AUCg	171.18	44.77	175.98	47.84	0.561
CRP	0.36	0.23	0.33	0.20	0.395
IL-6	0.47	0.18	0.48	0.16	0.723
IL-10	2.82	0.24	2.83	0.36	0.920
IL-1β	2.07	0.23	2.06	0.18	0.750
TNF-α	2.75	0.22	2.80	0.36	0.197

^**a**^Medications (IG, *n* = 86); *Biomarkers transformed (log/sqrt); Intervention Group (IG); Sham Control Group (SCG); mean (M); standard deviation (SD); Falls were standardized to a 12-month period to ensure comparability across assessment time points; Injurious falls were recorded as falls resulting in any injury; the type or severity of injury was not further specified; Falls Efficacy Scale-International (FES-I); Falls Efficacy Scale-International Avoidance Behavior (FES-IAB); Updated Perceived Control over Falling Scale (UP-CoF); ^**#**^UP-CoF (IG *n* = 71; SCG *n* = 67); Geriatric Anxiety Scale (GAS); Geriatric Depression Scale (GDS); Perceived Stress Scale 10 (PSS-10); EURQOL Visual Analogue Scale (EQ-5D VAS); Short Physical Performance Battery (SPPB); physical activity (PA); cortisol and amylase awakening response (CAR, AAR); area under the curve with respect to the ground (AUCg); C-reactive protein (CRP); Interleukin 6 (IL-6); Interleukin 10 (IL-10); Interleukin 1 beta (IL-1β); tumor necrosis factor alpha (TNF-α); body mass index (BMI)

### Longitudinal effects

Longitudinal changes across three time points were analyzed with linear mixed models (T0, T1, T2). Domain-level summaries are provided in Table 2 (Panel A) as an aggregate of outcome-level p values, whereas inferential reporting focuses on the FDR-adjusted outcome-level results (Panel B). Results are shown with Benjamini-Hochberg-FDR corrected p values. For psychological and functional outcomes as well as biomarkers only selected time effects were significant, whereas group effects and interaction (Time x Group) were non-significant. For anthropometrics, no effects (Time, Group, Time x Group) were significant. Time effects are presented in detail in the following.

**Table 2:** Significant domain-level and outcome-level effects (FDR-adjusted)

Panel A. Domain-level evidence					
Domain	Effect	*N* outcomes	*n* significant outcomes (FDR)	ACAT *p* (raw)	ACAT *p* (adjusted)
Psychological outcomes	Group	7	0	0.917	0.917
	Time	7	5	**< 0.001**	**< 0.001**
	Group × Time	7	0	0.121	0.241
Functional outcomes	Group	10	0	0.832	0.917
	Time	10	4	**< 0.001**	**< 0.001**
	Group × Time	10	0	0.091	0.241
Biomarkers (saliva + blood)	Group	11	0	0.594	0.917
	Time	11	5	**< 0.001**	**< 0.001**
	Group × Time	11	0	0.915	0.950
Anthropometrics	Group	3	0	0.762	0.917
	Time	3	0	0.068	0.068
	Group × Time	3	0	0.950	0.950

Panel B. Univariate outcome-level effects					
Domain	Outcome	Effect	*p*(FDR)	Semi-partial *R*^2^	Significant time contrasts (estimate, *p*)
Psychological outcomes	FES-I	Time	**< 0.001**	0.060	T0-T1: 4.48, ***p*< .001** T0-T2: 3.85, ***p*< .001**
	FES-IAB	Time	**< 0.001**	0.035	T0-T1: 2.90, ***p*< .001** T0-T2: 2.12, ***p*= .001**
	UP-CoF	Time	**< 0.001**	0.065	T0-T1: -2.42, ***p*< .001** T0-T2: -1.68, ***p*< .001**
	GAS	Time	**< 0.001**	0.016	T0-T1: 2.02, ***p*= .002** T0-T2: 1.88, ***p*= .003**
	PSS	Time	**0.007**	0.012	T0-T1: 1.51, ***p*= .010** T0-T2: 1.46, ***p*= .010**
Functional outcomes	Handgrip strength (kg)	Time	**< 0.001**	0.015	T0-T1: -0.76, ***p =*.040** T1-T2: -1.67, ***p*< .001** T0-T2: -2.43, ***p*< .001**
	SPPB (total Score)	Time	**0.046**	0.004	T0-T2: -0.41, ***p =*.034**
	SPPB (speed [m/sec])	Time	**0.004**	0.008	T0-T1: -0.05, ***p*< .001**
	Steps (daily average)	Time	**0.044**	0.006	T0-T2: 526.04, ***p =*.026**
Biomarkers	Cortisol (AUCg)	Time	**< 0.001**	0.042	T0-T1: -0.58, ***p*= .021** T1-T2: 1.24, ***p =*.007** T0-T2: 0.66, ***p*= .017**
	Amylase (AUCg)	Time	**0.007**	0.013	T1-T2: -13.16, ***p =*.003** T0-T2: -10.07, ***p*= .013**
	IL-6	Time	**< 0.001**	0.025	T1-T2: -0.06, ***p =*.001** T0-T2: -0.06, ***p =*.002**
	IL-1β	Time	**< 0.001**	0.038	T0-T1: 0.05, ***p =*.002** T1-T2: -0.10, ***p <*.001** T0-T2: -0.05, ***p =*.004**
	TNF-α	Time	**< 0.001**	0.040	T1-T2: 0.09, ***p*< .001** T0-T2: 0.09, ***p*< .001**

Panel A reports domain-level p values obtained by combining outcome-level p values within each domain using ACAT; ACAT combines multiple, potentially dependent p values into a single domain-level p value; p values are BH-adjusted across domains separately for each effect. N significant outcomes (FDR) indicate how many outcomes within the domain met the pre-specified outcome-level FDR threshold (*p* < .05) for the respective effect. Although all outcomes were analyzed, Panel B displays only statistically significant results. Panel B lists outcomes with FDR-significant effects; p(FDR) are BH-adjusted within domain × effect (across outcomes). Semi-partial R² indicates the unique variance explained by the respective effect. Post-hoc time-point contrasts are based on estimated marginal means (emmeans), with estimates computed as emmean (Time1) – emmean (Time2), and BH-adjusted within each contrast family. Falls Efficacy Scale-International (FES-I) score; Falls Efficacy Scale-International Avoidance Behavior (FES-IAB); Updated Perceived Control over Falling Scale (UP-CoF); Geriatric Anxiety Scale (GAS); Perceived Stress Scale 10 (PSS-10); Short Physical Performance Battery (SPPB); cortisol and amylase awakening response (CAR, AAR); area under the curve with respect to the ground (AUCg); Interleukin 6 (IL-6); Interleukin 1 beta (IL-1β); tumor necrosis factor alpha (TNF-α)

### Psychological outcomes

Within the psychological outcomes, FES-I, FES-IAB, UP-CoF, GAS and PSS showed a significant main effect of time (p(FDR) < 0.001, for PSS p(FDR) = 0.007), whereas GDS and EQ-5D VAS showed no significant time effects. Negative estimates indicate higher values at the later time point and vice versa. For time-point T0-T1 BH-adjusted post-hoc contrasts indicating differences for FES-I (estimate = 4.48, *p* < .001), FES-IAB (estimate = 2.9, *p* < .001), UP-CoF (estimate = − 2.42, *p* < .001), GAS (estimate = 2.02, *p* = .002) and PSS (estimate = 1.51, *p* = .010), indicating a reduction in FES-I, FES-IAB, GAS and PSS and an increase in UP-CoF scores.

For time-point T1-T2 none of the psychological outcomes (FES-I, FES-IAB, UP-CoF, GAS, GDS, PSS and EQ-5D VAS) showed significant change in BH-adjusted post-hoc contrasts, indicating that the improvement achieved immediately after the intervention remained largely stable until the follow-up.

**Fig. 1:**
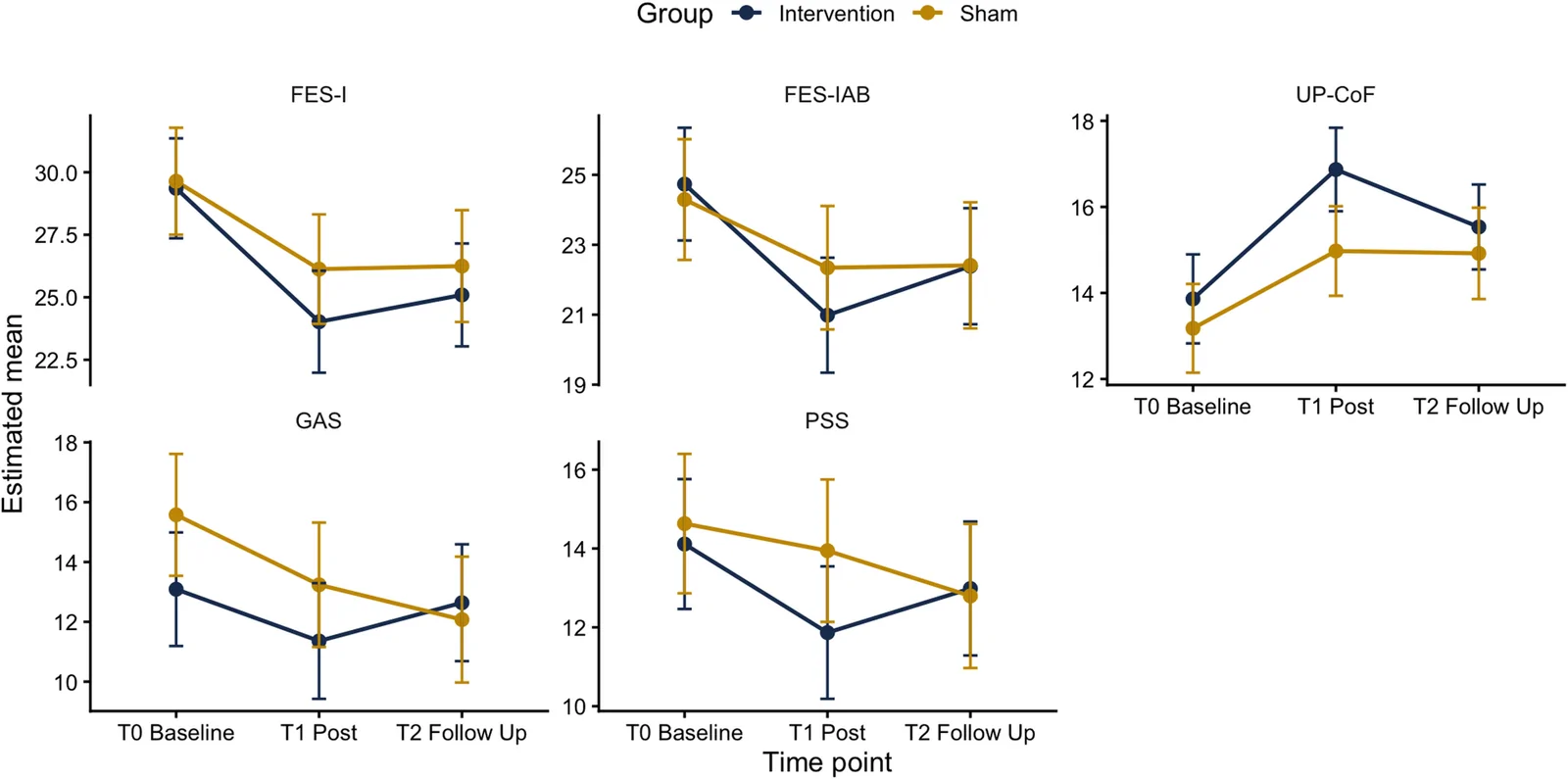
Change of psychological outcomes over all time points (T0, T1, T2). **Note.** Error bars indicate 95% confidence intervals; Falls Efficacy Scale-International (FES-I) score; Falls Efficacy Scale-International Avoidance Behavior (FES-IAB); Updated Perceived Control over Falling Scale (UP-CoF); Geriatric Anxiety Scale (GAS); Perceived Stress Scale 10 (PSS-10)

For time-point T0-T2 BH-adjusted post-hoc contrasts indicating differences for FES-I (estimate = 3.85, *p* < .001), FES-IAB (estimate = 2.12, *p* = .001), UP-CoF (estimate = − 1.68, *p* < .001), GAS (estimate = 1.88, *p* = .003) and PSS (estimate = 1.46, *p* = .010), indicating a reduction in FES-I, FES-IAB, GAS and PSS and an increase in UP-CoF scores across the entire study period. The change of all psychological outcomes over all time points (T0, T1, T2) is shown in Fig. 1.

### Functional outcomes

Within the functional outcomes, handgrip strength, SPPB total score, gait speed, determined as part of SPPB and step count showed a significant main effect of time (p(FDR) < 0.001, for SPPB total score p(FDR) = 0.046, gait speed p(FDR) = 0.004 and step count p(FDR) = 0.044), whereas other subscales of the SPPB total score, SPPB chair rising speed, SPPB-Balance, and PA level showed no significant time effects. Negative estimates indicate higher values at the later time point and vice versa. For time-point T0-T1 BH-adjusted post-hoc contrasts indicating differences for handgrip strength (estimate = -0.76, *p* = .040) and gait speed (estimate = -0.05, *p* < .001), indicating an improvement in handgrip strength and gait speed (near the threshold of clinical relevance).

For time-point T1-T2 BH-adjusted post-hoc contrasts indicating differences for handgrip strength (estimate = -1.67, *p* < .001), indicating a further improvement in handgrip-strength until the follow-up time point.

**Fig. 2:**
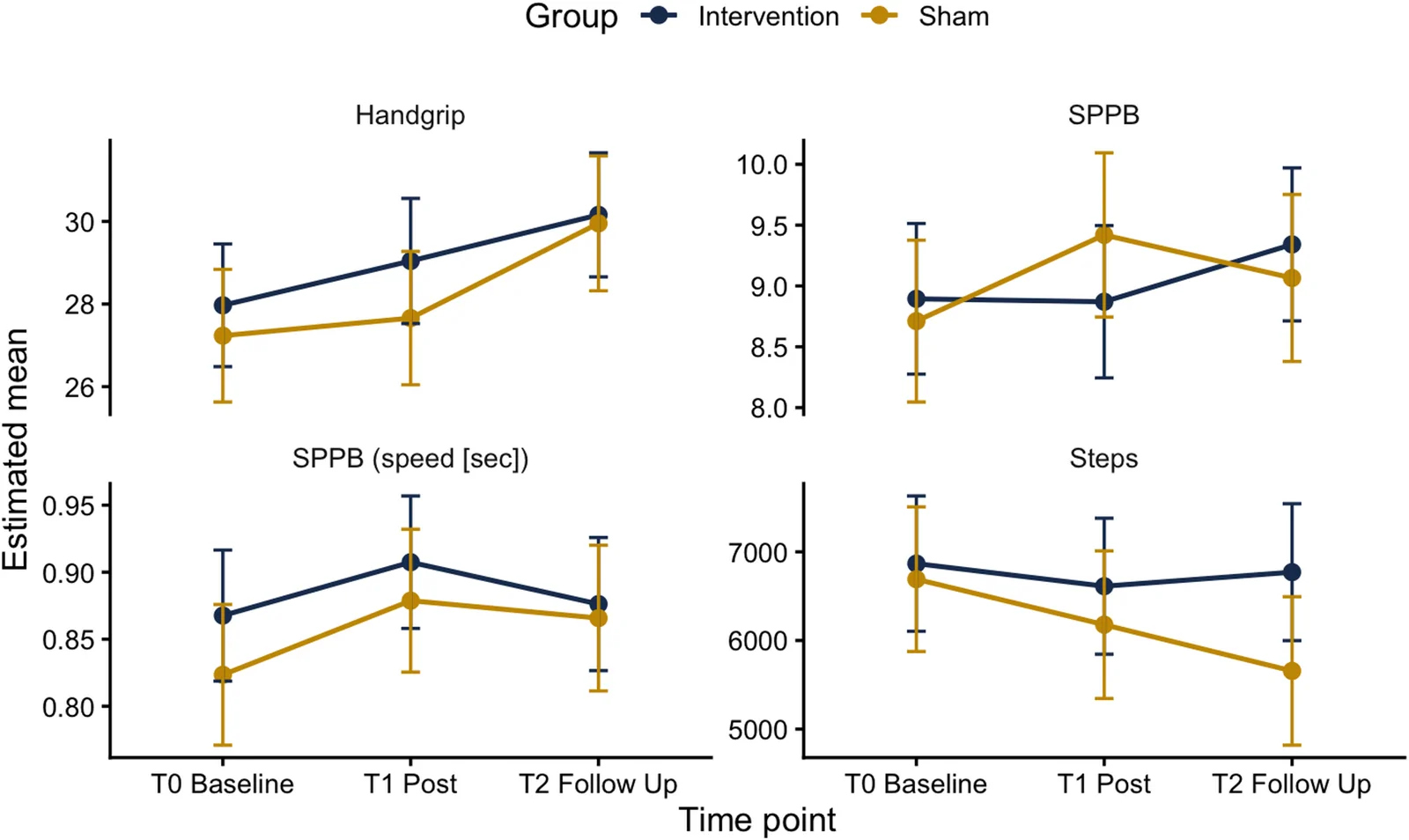
Change of functional outcomes over all time points (T0, T1, T2). **Note.** Error bars indicate 95% confidence intervals; Short Physical Performance Battery (SPPB)

For time-point T0-T2 BH-adjusted post-hoc contrasts indicating differences for handgrip strength (estimate = -2.43, *p* < .001), SPPB total score (estimate = -0.41, *p* = .034) and step count (estimate = 526.04, *p* = .026), indicating an improvement in handgrip strength and SPPB total score, while a decline of step count was observed over the entire study period. The change of all functional outcomes over all time points (T0, T1, T2) is shown in Fig. 2.

### Stress system activity and inflammation

Within the salivary and blood biomarkers, Cortisol AUCg, Amylase AUCg, IL-6, IL-1β and TNF-α showed a significant main effect of time (p(FDR) < 0.001, for Amylase AUCg p(FDR) = 0.007), whereas CAR, Cortisol Slope, AAR, Amylase Slope, CRP and IL-10 showed no significant time effects. Negative estimates indicate higher values at the later time point and vice versa. For time-point T0-T1 BH-adjusted post-hoc contrasts indicating differences for Cortisol AUCg (estimate = -0.58, *p* = .021) and IL-1β (estimate = 0.05, *p* = .002), indicating an increase of total diurnal cortisol output and a reduction in IL-1β. Amylase AUCg, IL-6 and TNF-α remained stable, suggesting no time effect on these biomarkers.

For time-point T1-T2 BH-adjusted post-hoc contrasts indicating differences for Cortisol AUCg (estimate = 1.24, *p* = .007), Amylase AUCg (estimate = -13.16, *p* = .003), IL-6 (estimate = -0.06, *p* = .001), IL-1β (estimate = -0.10, *p* = < .001) and TNF-α (estimate = 0.09, *p* < .001), suggesting a reduction of total diurnal cortisol output and TNF-α, and an increase in total diurnal amylase output, IL-6 and IL-1β over time.

For time-point T0-T2 BH-adjusted post-hoc contrasts indicating differences for Cortisol AUCg (estimate = 0.66, *p* < .017), Amylase AUCg (estimate = -10.07, *p* = .013), IL-6 (estimate = -0.06, *p* = .002), IL-1β (estimate = -0.05, *p* = .004) and TNF-α (estimate = 0.09, *p* < .001), suggesting a reduction in total diurnal cortisol output and TNF-α and an increase of total diurnal amylase output, IL-6 and IL-1β across the entire study period.

**Fig. 3:**
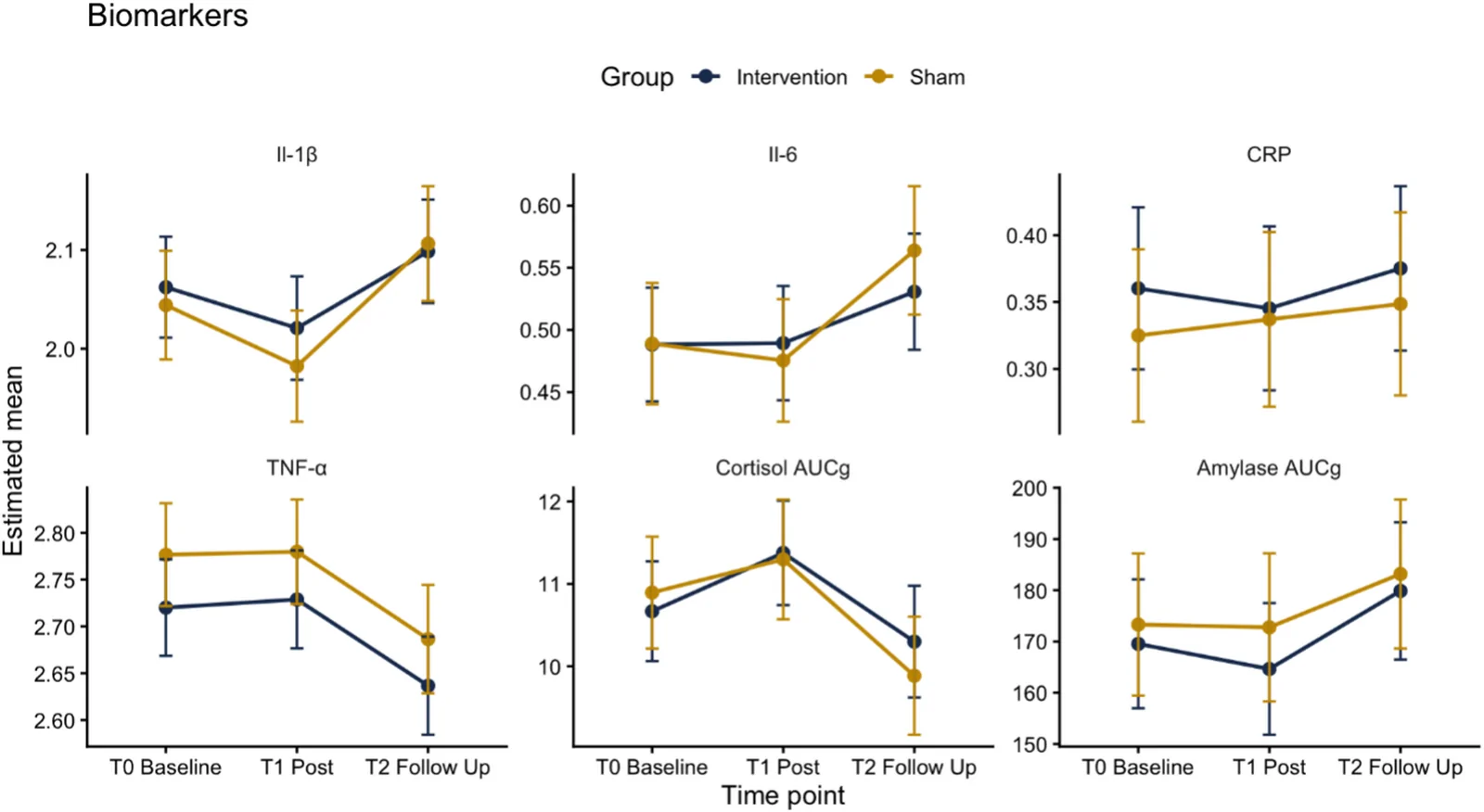
Change of stress system activity and inflammation over all time points. **Note**. Error bars indicate 95% confidence intervals; biomarkers are shown on transformed scales; Interleukin 1 beta (IL-1β); Interleukin 6 (IL-6); C-reactive protein (CRP); tumor necrosis factor alpha (TNF-α); cortisol and amylase area under the curve with respect to the ground (AUCg)

Inflammatory cytokine changes present an inconsistent pattern, with respect to time point and direction. The change of all stress system activity and inflammation over all time points (T0, T1, T2) is shown in Fig. 3.

### Anthropometric outcomes

Anthropometric variables, BMI, skeletal muscle mass and skeletal muscle index showed no significant time effect (Table 2 Panel A).

## Discussion

This longitudinal analysis of FEARFALL, characterized changes on psychological, functional, pathophysiological and anthropometric dimensions in older adults with concerns about falling. Contrary to our expectation no differences between the intervention (IG) and sham control group (SCG) were found. CaF, perceived stress and anxiety declined, handgrip strength and selected physical performance measures improved over time, total diurnal cortisol output decreased, and total diurnal amylase output increased, and pro-inflammatory cytokine changes present an inconsistent picture (IL-6 and IL-1β increased, TNF-α decreased). Primary per-protocol analysis were corroborated by intention-to-treat (ITT) analysis underscoring the robustness of our findings (see Supplementary Information 4).

The impact of interventions, particularly cognitive behavioral therapy (CBT) with exercise programs, on reducing fall-related concerns in older adults, has been explored in two systematic reviews [20, 21]. In the study presented here, a combined cognitive behavioral (based on “A Matter of Balance” (AMB)) [41] and exercise intervention [23] resulted in no differences between groups immediately after the intervention [22] and after 8 months of follow-up compared with the SCG regarding CaF reduction. In contrast to studies mentioned above using placebo control or usual care [21, 42, 43], we integrated a SCG. Lenouvel et al. (2023) carried out subgroup analysis comparing placebo control and usual care without any change of the results, however differing from our SCG [21]. Equivalent session frequency and study personnel / participants contact across groups may have accounted for the effects, yielding no meaningful between-groups differences. Nevertheless, because social support, frequent social engagement, and greater opportunities for physical activity have been identified as protective factors against CaF [44], our SCG cannot be considered equivalent to the multicomponent intervention. Therefore, our SCG may be more appropriately interpreted as an active control group, which could explain the null between-group findings. In addition, the SCG was intentionally designed to match the intervention group in session frequency and duration, which supports the assumption that it functioned as an active control group rather than an inactive control. Another potential explanation is that the intensity and duration of the multicomponent intervention was insufficient to detect between-group differences in this comparatively fit cohort.

Both groups showed beneficial changes over time in different dimensions. Longitudinal reduction in CaF and activity avoidance and an increase in perceived control were identified, in accordance to other AMB studies as described above [21, 42, 43]. One single study reported on anxiety using CBT intervention showing inconclusive effects [45]. We observed perceived stress and anxiety reduction, which might be explained by CaF reduction, potentially contributing to prevent loss of independence [46] and social isolation [47]. Another contributing factor over time could be the increase in the UP-CoF scores, demonstrating a higher perceived control over falling, and thus decreasing CaF. In contrast, low perceived control over falling could decrease physical activity, and increase fall risk by maladaptive behavior [28]. Our findings suggest that assessing additional psychological constructs (e.g. anxiety, perceived control over falling) is necessary to achieve a more comprehensive understanding of CaF. CaF also negatively affect physical activity resulting in reduced activity and declining muscle mass and function (sarcopenia) and enhanced CaF [6]. Nicklen recently reported a bidirectional relationship of CaF and frailty [48], showing that frailty can be both cause and consequence of CaF by activity restriction. Modest improvements in functional outcomes were observed, with an increase in SPPB total score at long-term follow-up and a post-intervention increase in SPPB gait speed (m/s) that was sustained at long-term follow-up, potentially reflecting an attenuation of the vicious cycle of functional decline. In the SPRINTT study [49], a multicomponent intervention study, participants with an SPPB score of 8 or 9 showed a 0.5-point increase in SPPB score at 36 months, regardless of group allocation. In the study presented here both groups improved by 0.41 points within one year. This observation indicates comparable results for both studies not exceeding marginal clinically meaningful changes for SPPB, described by changes > 1.0 point [50, 51]. Gait speed, as part of the SPPB score, improved immediately after the intervention by 0.05 m/s which is near the threshold of clinical relevance (0.1 m/s) without further change.

Handgrip strength is frequently employed as an indicator of current health status in sarcopenia research [52, 53]. Multicomponent interventions in older adults have typically resulted in slight to moderate improvements in handgrip strength, without consistent evidence of parallel gains in muscle mass [54, 55]. Although handgrip strength increased over the overall study period in this analysis, the observed effect must be interpreted with caution. While the gain in handgrip strength could be interpreted as an indication of functional improvement, remaining below the estimateds clinical relevance (5.0 to 6.5 kg) [56], a habituation effect regarding the measurement method cannot be ruled out.

Countering the potential improvement in functional measurements, step count remained stable after the intervention and even decreased over the entire study period, which could indicate a possible decline in daily physical activity.

We examined whether CaF are reflected in stress-related biomarkers. Although CaF are not formally classified as a stress condition, they exhibit common stress-response elements, and may engage central stress pathways [10, 12, 14, 57]. Total diurnal output of cortisol reflects basal activity of the HPA-axis, whereas total diurnal output of salivary α-amylase is considered a marker of basal SNS activity [37]. Whereas several exercise as well as CBT studies have examined the change of neuroendocrine markers [58–60] with reduced or increased cortisol concentrations and no change of amylase, we observed decreased total diurnal output of cortisol and increased total diurnal output of α-amylase. Despite the differences in the target population (regarding age and disease profile), these findings could be an indication of an altered reaction of the stress systems. Functioning CAR and slope, a small post-intervention increase in total diurnal cortisol in both groups, likely reflects modest engagement, followed by long-term decline of HPA axis activation which may indicate adaptation or partial normalization of basal HPA activity. In contrast, total diurnal α-amylase increased, suggesting higher SNS axis activity; whether this reflects adaptive normalization or heightened reactivity remains uncertain.

Neuroendocrine shifts are relevant for immune regulation. Anxiety is associated with elevated CRP and peripheral cytokine levels [61–63] and predicts an increased inflammatory response to acute stress [64–66], thus, even non-clinical manifestations of anxiety and fear significantly influence the immune response [67]. Inflammatory marker changes in our study present a non-cohesive picture, as CRP and IL-10 remained stable, IL-6 and IL-1β rise, and TNF-α decreases, warranting interpretation with caution, as cytokine profiles are influenced by multiple factors. The increase in IL-6 might be interpreted as an increase in overall inflammatory activity with implications for inflammaging and age-associated morbidity [68], though it could also reflect age-appropriate elevations that appeared attenuated immediately post-intervention or might be partly related to the observed decline in total diurnal cortisol output. An adaptive IL-6 response to higher physical activity seems less likely, as the daily step count decreased.

Moreover, the lack of significant changes in anthropometric variables observed here may suggest maintenance of anthropometric status over time. Finally, the multicomponent intervention proved to be feasible and safe in vulnerable older adults with some functional limitations.

This study has several limitations. First, due to the inclusion criteria of FEARFALL, only people with moderate or high CaF were included in this study, which does not allow any conclusions to be drawn about people without CaF. Furthermore, older persons living in the community without significant cognitive or neurological impairments were included to ensure safe and full participation, limiting generalizability. The variability of results or absence of significant changes may reflect the heterogeneity of community-dwelling older adults with CaF and may partly explain the different responses to the interventions. Given the deviation from the planned sample size (*N* = 200) and the statistical power being geared towards T1, the absence of significant Group × Time interactions, particularly at long-term follow-up, should be interpreted with caution, as small interaction effects may have remained undetected. Another potential limitation is that the Hawthorne effect cannot be excluded, as participants’ awareness of observation may have influenced their responses, and unmeasured confounding factors such as dietary habits and the assessment of frailty may have also contributed to a distortion of the results.

Despite its limitations, the strengths of our study include a longitudinal design of a randomized controlled trial with rigorous selection criteria yielding a well-defined cohort of community-dwelling adults ≥ 70 years. Compliance with the intervention programs was very high in both groups (> 77% participation), which compares favorably to other exercise programs aimed at reducing CaF [20].

The collection of multiple saliva samples across consecutive days improving biomarker reliability. Moreover, integrating psychological, functional, physiological and anthropometric measures allowed for a multidimensional analysis of the research questions.

Future studies should consider including control groups with equal contact frequencies as intervention groups to investigate the effectiveness of multicomponent intervention programs. Future research is required to carefully select populations that might exhibit different responses to more individualized interventions over an extended period. This will provide a more comprehensive understanding of the sustainability and efficacy of CaF reduction strategies.

## Conclusions

In the randomized controlled FEARFALL trial, the intervention did not confer additional benefits over the sham control condition. Both groups showed similar time-related improvements in CaF, and changes in basal stress-system activity and inflammatory cytokines were likewise comparable. To achieve additional long-term benefits, future work should intensify and further tailor multidimensional interventions that integrate individual, psychological, functional, and pathophysiological aspects to promote healthy aging and mitigate the consequences of falls.

## Supplementary Information

Below is the link to the electronic supplementary material.

Supplementary Material 1

Supplementary Material 2

Supplementary Material 3

Supplementary Material 4

## Data Availability

The datasets generated and examined during this study are not available to the public, but can be obtained by contacting the corresponding author.
