# Supplementary material for: Reducing concerns about falling in older adults: evaluation of a multi-component intervention and the role of stress and inflammation markers - long-term effects of the FEARFALL randomized controlled trial
Source: Aging Clin Exp Res. 2026 Sep 19;38(1):195. doi: 10.1007/s40520-026-03515-z (PMC13597564; doi:10.1007/s40520-026-03515-z)
Supplement: Supplementary file 2 [file 40520_2026_3515_MOESM2_ESM.docx]

**Reducing Concerns about Falling in Older Adults: Effectiveness of a multi-component intervention and the role of stress and inflammation markers - Long-term effects of the FEARFALL randomized controlled trial**

Sabine Britting^1#^, Anja Müller^2#^, Robert Kob^1^, Ellen Freiberger^1^, Nicolas Rohleder^2^

^#^*shared first author*

^1^ Institute for Biomedicine of Aging, Department of Internal Medicine-Geriatrics, Friedrich-Alexander-Universität Erlangen-Nürnberg, Nuremberg, Germany

^2^ Chair of Health Psychology, Department of Psychology, Friedrich-Alexander-Universität Erlangen-Nürnberg, Erlangen, Germany

**Corresponding author**

Sabine Britting, Kobergerstr. 60, 90408 Nuremberg, Germany

Email: sabine.britting@fau.de

**ORCID**

Sabine Britting	0000-0001-8237-6105 23

Anja Müller	0009-0004-4101-9178 28

Robert Kob	0000-0002-0406-092X

Ellen Freiberger	0000-0002-6506-6276 26

Nicolas Rohleder	0000-0003-2602-517X 2

**Supplementary Information 4. Complete domain-level and outcome-level mixed-model results and BH-adjusted contrasts (intention-to-treat analysis).**

**Panel A. Domain-level evidence.**

| **Domain** | **Effect** | **N outcomes** | **n significant outcomes (FDR)** | **ACAT *p* (raw)** | **ACAT *p* (adjusted)** |
| --- | --- | --- | --- | --- | --- |
| Psychological outcomes | Group | 7 | 0 | .221 | .659 |
|  | Time | 7 | 5 | **< .001** | **< .001** |
|  | Group × Time | 7 | 0 | .121 | .241 |
| Functional outcomes | Group | 10 | 0 | .832 | .917 |
|  | Time | 10 | 5 | **< .001** | **< .001** |
|  | Group × Time | 10 | 0 | .091 | .241 |
| Biomarkers (saliva + blood) | Group | 11 | 0 | .594 | .917 |
|  | Time | 11 | 5 | **< .001** | **< .001** |
|  | Group × Time | 11 | 0 | .915 | .950 |
| Anthropometrics | Group | 3 | 0 | .762 | .917 |
|  | Time | 3 | 0 | .068 | .068 |
|  | Group × Time | 3 | 0 | .950 | .950 |

**Panel B. Univariate outcome-level effects.**

| **Domain** | **Outcome** | **Effect** | ***p*(FDR)** | **Semi-partial *R^2^*** | **Contrasts (estimate, BH-adjusted *p*)** |
| --- | --- | --- | --- | --- | --- |
| Psychological outcomes | FES-I | Group | 0.753 | 0.001 | T0: Intervention-Sham: 0.35, *p* = .781  T1: Intervention-Sham: -1.33, *p* = .320 T2: Intervention-Sham: -0.65, *p* = .635 |
|  |  | Time | **< .001** | 0.059 | T0-T1: 4.31, ***p* < .001**  T1-T2: -0.36, *p* = .602  T0-T2: 3.94, ***p* < .001** |
|  |  | Group × Time | 0.614 | 0.002 | Intervention:  T0-T1: 5.07, ***p* < .001**  T1-T2: -0.67, *p* = .479  T0-T2: 4.40, ***p* < .001**  Sham:  T0-T1: 3.38, ***p* = .001**  T1-T2: 0.01, *p* = .992  T0-T2: 3.39, ***p* = .001** |
|  | FES-IAB | Group | 0.753 | 0.001 | T0: Intervention-Sham: 0.01, *p* = .991  T1: Intervention-Sham: -1.04, *p* = .341  T2: Intervention-Sham: -0.14, *p* = .901 |
|  |  | Time | **< .001** | 0.030 | T0-T1: 2.56, ***p* < .001**  T1-T2: -0.43, *p* = .463  T0-T2: 2.13, ***p* < .001** |
|  |  | Group × Time | 0.614 | 0.001 | Intervention:  T0-T1: 3.03, ***p* < .001**  T1-T2: -0.83, *p* = .290  T0-T2: 2.20, ***p* = .007**  Sham:  T0-T1: 1.98, ***p* = .026**  T1-T2: 0.07, *p* = .937  T0-T2: 2.05, ***p* = .026** |
|  | UP-CoF | Group | 0.753 | 0.003 | T0: Intervention-Sham: -1.58, *p* = .179  T1: Intervention-Sham: -1.32, *p* = .284  T2: Intervention-Sham: 0.74, *p* = .557 |
|  |  | Time | 0.003 | 0.011 | T0-T1: 1.68, ***p* = .006**  T1-T2: -0.14, *p* = .799  T0-T2: 1.53, ***p* = .009** |
|  |  | Group × Time | 0.507 | 0.004 | Intervention:  T0-T1: 1.56, *p* = .095  T1-T2: -1.09, *p* = .225  T0-T2: 0.47, *p* = .531  Sham:  T0-T1: 1.82, ***p* = .034**  T1-T2: 0.97, *p* = .239  T0-T2: 2.79, ***p* = .002** |
|  | GAS | Group | 0.359 | 0.013 | T0: Intervention-Sham: -0.61, *p* = .062  T1: Intervention-Sham: -0.50, *p* = .144  T2: Intervention-Sham: -0.26, *p* = .465 |
|  |  | Time | 0.072 | 0.006 | T0-T1: 0.36, *p* = .080  T1-T2: -0.09, *p* = .571  T0-T2: 0.26, *p* = .168 |
|  |  | Group × Time | 0.614 | 0.001 | Intervention:  T0-T1: 0.31, *p* = .471  T1-T2: -0.21, *p* = .543  T0-T2: 0.10, *p* = .650  Sham:  T0-T1: 0.42, *p* = .124  T1-T2: 0.04, *p* = .873  T0-T2: 0.46, *p* = .124 |
|  | GDS | Group | 0.359 | 0.013 | T0: Intervention-Sham: -1.49, *p* = .605  T1: Intervention-Sham: -2.04, *p* = .503  T2: Intervention-Sham: -4.15, *p* = .181 |
|  |  | Time | 0.072 | 0.006 | T0-T1: -2.31, *p* = .153  T1-T2: 0.00, *p* = .998  T0-T2: -2.30, *p* = .153 |
|  |  | Group × Time | 0.614 | 0.001 | Intervention:  T0-T1: -2.06, *p* = .616  T1-T2: 0.98, *p* = .616  T0-T2: -1.08, *p* = .616  Sham:  T0-T1: -2.60, *p* = .305  T1-T2: -1.14, *p* = .592  T0-T2: -3.74, *p* = .218 |
|  | PSS | Group | 0.907 | 0.000 | T0: Intervention-Sham: 0.39, *p* = .709  T1: Intervention-Sham: -1.22, *p* = .271  T2: Intervention-Sham: 0.50, *p* = .658 |
|  |  | Time | **0.020** | 0.009 | T0-T1: 1.20, ***p* = .020**  T1-T2: 0.10, *p* = .846  T0-T2: 1.30, ***p* = .020** |
|  |  | Group × Time | 0.507 | 0.003 | Intervention:  T0-T1: 1.93, ***p* = .009**  T1-T2: -0.68, *p* = .314  T0-T2: 1.25, *p* = .094  Sham:  T0-T1: 0.32, *p* = .655  T1-T2: 1.03, *p* = .245  T0-T2: 1.35, *p* = .189 |
|  | Lifequal  (EQ-5D VAS) | Group | 0.251 | 0.016 | T0: Intervention-Sham: 0.64, *p* = .316  T1: Intervention-Sham: 1.56, ***p* = .017**  T2: Intervention-Sham: 0.73, *p* = .275 |
|  |  | Time | **< .001** | 0.058 | T0-T1: -2.19, ***p* < .001**  T1-T2: 0.44, *p* = .259  T0-T2: -1.75, ***p* < .001** |
|  |  | Group × Time | 0.614 | 0.003 | Intervention:  T0-T1: -2.62, ***p* < .001**  T1-T2: 0.82, *p* = .125  T0-T2: -1.80, ***p* = .002**  Sham:  T0-T1: -1.71, ***p* = .005**  T1-T2: -0.01, *p* = .989  T0-T2: -1.71, ***p* = .005** |
| Functional outcomes | Handgrip strength (kg) | Group | 0.940 | 0.001 | T0: Intervention-Sham: 0.38, *p* = .682  T1: Intervention-Sham: 1.10, *p* = .252  T2: Intervention-Sham: 0.06, *p* = .948 |
|  |  | Time | **< .001** | 0.015 | T0-T1: -0.69, ***p* = .046**  T1-T2: -1.67, ***p* < .001**  T0-T2: -2.36, ***p* < .001** |
|  |  | Group × Time | 0.389 | 0.001 | Intervention:  T0-T1: -1.02, ***p* = .029**  T1-T2: -1.19, ***p* = .021**  T0-T2: -2.21**, *p* < .001**  Sham:  T0-T1: -0.31, *p* = .546  T1-T2: -2.22, ***p* < .001**  T0-T2: -2.53, ***p* < .001** |
|  | PA-Level | Group | 0.940 | 0.000 | T0: Intervention-Sham: 0.07, *p* = .537  T1: Intervention-Sham: 0.05, *p* = .689  T2: Intervention-Sham: -0.06, *p* = .629 |
|  |  | Time | 0.028 | 0.009 | T0-T1: -0.11, ***p* = .048**  T1-T2: 0.16, ***p* = .013**  T0-T2: 0.05, *p* = .408 |
|  |  | Group × Time | 0.489 | 0.001 | Intervention:  T0-T1: -0.10, *p* = .171  T1-T2: 0.21, ***p* = .020**  T0-T2: 0.10, *p* = .171  Sham:  T0-T1: -0.13, *p* = .308  T1-T2: 0.10, *p* = .308  T0-T2: -0.02, *p* = .787 |
|  | SPPB  (total Score) | Group | 0.940 | 0.000 | T0: Intervention-Sham: 0.18, *p* = .649  T1: Intervention-Sham: -0.59, *p* = .144  T2: Intervention-Sham: 0.18, *p* = .668 |
|  |  | Time | **0.015** | 0.005 | T0-T1: -0.36, ***p* = .020**  T1-T2: -0.06, *p* = .676  T0-T2: -0.42, ***p* = .015** |
|  |  | Group × Time | 0.091 | 0.005 | Intervention:  T0-T1: -0.01, *p* = .941  T1-T2: -0.40, *p* = .065  T0-T2: -0.41, *p* = .065  Sham:  T0-T1: -0.79, ***p* < .001**  T1-T2: 0.37, *p* = .100  T0-T2: -0.42, *p* = .088 |
|  | SPPB (balance) | Group | 0.940 | 0.007 | T0: Intervention-Sham: 0.24, ***p* = .026**  T1: Intervention-Sham: 0.02, *p* = .839  T2: Intervention-Sham: 0.05, *p* = .683 |
|  |  | Time | 0.057 | 0.003 | T0-T1: -0.10, *p* = .074  T1-T2: 0.05, *p* = .346  T0-T2: -0.06, *p* = .328 |
|  |  | Group × Time | 0.132 | 0.004 | Intervention:  T0-T1: -0.00, *p* = .958  T1-T2: 0.03, *p* = .934  T0-T2: 0.03, *p* = .934  Sham:  T0-T1: -0.23, ***p* = .003**  T1-T2: 0.06, *p* = .413  T0-T2: -0.17, ***p* = .027** |
|  | SPPB (4m [score 1-4]) | Group | 0.940 | 0.000 | T0: Intervention-Sham: 0.24, *p* = .310  T1: Intervention-Sham: -0.44, *p* = .108  T2: Intervention-Sham: 0.15, *p* = .715 |
|  |  | Time | 0.211 | 0.008 | T0-T1: 0.07, *p* = .617  T1-T2: -0.42, *p* = .119  T0-T2: -0.35, *p* = .119 |
|  |  | Group × Time | 0.132 | 0.009 | Intervention:  T0-T1: 0.38, *p* = .064  T1-T2: -0.66, ***p* = .030**  T0-T2: -0.28, *p* = .254  Sham:  T0-T1: -0.30, *p* = .375  T1-T2: -0.07, *p* = .825  T0-T2: -0.38, *p* = .375 |
|  | SPPB (4m speed [m/sec]) | Group | 0.940 | 0.003 | T0: Intervention-Sham: 0.04, *p* = .189  T1: Intervention-Sham: 0.02, *p* = .547  T2: Intervention-Sham: 0.01, *p* = .865 |
|  |  | Time | **0.014** | 0.006 | T0-T1: -0.04, ***p* = .003**  T1-T2: 0.03, *p* = .053  T0-T2: -0.01, *p* = .273 |
|  |  | Group × Time | 0.389 | 0.001 | Intervention:  T0-T1: -0.03, *p* = .092  T1-T2: 0.03, *p* = .092  T0-T2: 0.00, *p* = .901  Sham:  T0-T1: -0.05, ***p* = .012**  T1-T2: 0.02, *p* = .329  T0-T2: -0.03, *p* = .113 |
|  | SPPB (chair rise [score 1-4] | Group | 0.940 | 0.000 | T0: Intervention-Sham: 0.01, *p* = .954  T1: Intervention-Sham: -0.26, *p* = .233  T2: Intervention-Sham: 0.08, *p* = .715 |
|  |  | Time | 0.056 | 0.005 | T0-T1: -0.23, ***p* = .047**  T1-T2: 0.11, *p* = .282  T0-T2: -0.12, *p* = .282 |
|  |  | Group × Time | 0.302 | 0.003 | Intervention:  T0-T1: -0.11, *p* = .572  T1-T2: -0.04, *p* = .756  T0-T2: -0.15, *p* = .572  Sham:  T0-T1: -0.38, ***p* = .022**  T1-T2: 0.30, *p* = .069  T0-T2: -0.08, *p* = .571 |
|  | SPPB (chair rise [sec]) | Group | 0.940 | 0.000 | T0: Intervention-Sham: -1.06, *p* = .399  T1: Intervention-Sham: 0.45, *p* = .742  T2: Intervention-Sham: 1.90, *p* = .192 |
|  |  | Time | 0.153 | 0.012 | T0-T1: -0.33, *p* = .719  T1-T2: -1.66, *p* = .140  T0-T2: -1.99, *p* = .114 |
|  |  | Group × Time | 0.389 | 0.006 | Intervention:  T0-T1: -1.02, *p* = .410  T1-T2: -2.31, *p* = .123  T0-T2: -3.33, *p* = .031  Sham:  T0-T1: 0.48, *p* = .793  T1-T2: -0.86, *p* = .793  T0-T2: -0.37, *p* = .793 |
|  | Steps (daily average) | Group | 0.940 | 0.003 | T0: Intervention-Sham: 108.55, *p* = .834  T1: Intervention-Sham: 179.76, *p* = .735  T2: Intervention-Sham: 860.15, *p* = .111 |
|  |  | Time | **0.021** | 0.006 | T0-T1: 351.88, *p* = .087  T1-T2: 194.48, *p* = .310  T0-T2: 546.37, ***p* = .013** |
|  |  | Group × Time | 0.221 | 0.003 | Intervention:  T0-T1: 316.26, *p* = .602  T1-T2: -112.35, *p* = .661  T0-T2: 203.91, *p* = .639  Sham:  T0-T1: 387.47, *p* = .162  T1-T2: 568.04, *p* = .071  T0-T2: 955.51, *p* = .002 |
|  | Falls | Group | 0.940 | 0.000 | T0: Intervention-Sham: -0.01, *p* = .862  T1: Intervention-Sham: 0.04, *p* = .380  T2: Intervention-Sham: -0.05, *p* = .299 |
|  |  | Time | 0.421 | 0.002 | T0-T1: -0.01, *p* = .766  T1-T2: 0.03, *p* = .401  T0-T2: 0.02, *p* = .401 |
|  |  | Group × Time | 0.221 | 0.004 | Intervention:  T0-T1: -0.03, *p* = .314  T1-T2: 0.07, ***p* = .047**  T0-T2: 0.04, *p* = .208  Sham:  T0-T1: 0.02, *p* = .835  T1-T2: -0.02, *p* = .835  T0-T2: 0.00, *p* = .963 |
| Biomarkers | CAR | Group | 0.567 | 0.006 | T0: Intervention-Sham: -0.00, *p* = .955  T1: Intervention-Sham: 0.03, *p* = .535  T2: Intervention-Sham: 0.10, ***p* = .025** |
|  |  | Time | 0.972 | 0.000 | T0-T1: -0.01, *p* = .919  T1-T2: 0.00, *p* = .919  T0-T2: -0.00, *p* = .919 |
|  |  | Group × Time | 0.763 | 0.010 | Intervention:  T0-T1: -0.02, *p* = .549  T1-T2: -0.03, *p* = .549  T0-T2: -0.05, *p* = .436  Sham:  T0-T1: 0.01, *p* = .810  T1-T2: 0.04, *p* = .442  T0-T2: 0.05, *p* = .442 |
|  | Cortisol Slope | Group | 0.681 | 0.005 | T0: Intervention-Sham: 0.00, *p* = .945  T1: Intervention-Sham: -0.00, *p* = .194  T2: Intervention-Sham: -0.00, *p* = .235 |
|  |  | Time | 0.972 | 0.001 | T0-T1: 0.00, *p* = .831  T1-T2: -0.00, *p* = .831  T0-T2: 0.00, *p* = .831 |
|  |  | Group × Time | 0.804 | 0.004 | Intervention:  T0-T1: 0.00, *p* = .448  T1-T2: -0.00, *p* = .865  T0-T2: 0.00, *p* = .448  Sham:  T0-T1: -0.00, *p* = .943  T1-T2: -0.00, *p* = .946  T0-T2: -0.00, *p* = .943 |
|  | Cortisol (AUCg) | Group | 0.737 | 0.003 | T0: Intervention-Sham: -0.00, *p* = .999  T1: Intervention-Sham: 0.10, *p* = .831  T2: Intervention-Sham: 0.73, *p* = .113 |
|  |  | Time | **< .001** | 0.039 | T0-T1: -0.59, ***p* = .014**  T1-T2: 1.21, ***p* < .001**  T0-T2: 0.62, ***p* = .014** |
|  |  | Group × Time | 0.797 | 0.005 | Intervention:  T0-T1: -0.63, *p* = .065  T1-T2: 0.92, ***p* = .022**  T0-T2: 0.29, *p* = .380  Sham:  T0-T1: -0.54, *p* = .143  T1-T2: 1.56, ***p* < .001**  T0-T2: 1.02, ***p* = .009** |
|  | AAR | Group | 0.567 | 0.009 | T0: Intervention-Sham: -0.34, *p* = .463  T1: Intervention-Sham: -0.26, *p* = .605  T2: Intervention-Sham: -0.98, *p* = .071 |
|  |  | Time | 0.816 | 0.002 | T0-T1: 0.02, *p* = .948  T1-T2: -0.27, *p* = .670  T0-T2: -0.25, *p* = .670 |
|  |  | Group × Time | 0.804 | 0.004 | Intervention:  T0-T1: -0.01, *p* = .976  T1-T2: 0.05, *p* = .976  T0-T2: 0.04, *p* = .976  Sham:  T0-T1: 0.07, *p* = .889  T1-T2: -0.67, *p* = .330  T0-T2: -0.60, *p* = .330 |
|  | Amylase Slope | Group | 0.737 | 0.001 | T0: Intervention-Sham: 0.01, *p* = .820  T1: Intervention-Sham: 0.02, *p* = .553  T2: Intervention-Sham: 0.01, *p* = .809 |
|  |  | Time | 0.816 | 0.003 | T0-T1: 0.00, *p* = .885  T1-T2: -0.02, *p* = .531  T0-T2: -0.02, *p* = .531 |
|  |  | Group × Time | 0.957 | 0.000 | Intervention:  T0-T1: -0.00, *p* = .914  T1-T2: -0.02, *p* = .840  T0-T2: -0.02, *p* = .840  Sham:  T0-T1: 0.01, *p* = .724  T1-T2: -0.03, *p* = .724  T0-T2: -0.02, *p* = .724 |
|  | Amylase (AUCg) | Group | 0.737 | 0.003 | T0: Intervention-Sham: -4.59, *p* = .579  T1: Intervention-Sham: -6.47, *p* = .454  T2: Intervention-Sham: -5.05, *p* = .569 |
|  |  | Time | **< .001** | 0.015 | T0-T1: 1.93, *p* = .550  T1-T2: -13.97, ***p* < .001**  T0-T2: -12.04, ***p* < .001** |
|  |  | Group × Time | 0.957 | 0.000 | Intervention:  T0-T1: 2.69, *p* = .523  T1-T2: -14.56, ***p* = .006**  T0-T2: -11.87, ***p* = .015**  Sham:  T0-T1: 0.81, *p* = .873  T1-T2: -13.14, ***p* = .030**  T0-T2: -12.33, ***p* = .030** |
|  | CRP | Group | 0.737 | 0.001 | T0: Intervention-Sham: 0.03, *p* = .499  T1: Intervention-Sham: 0.01, *p* = .798  T2: Intervention-Sham: 0.01, *p* = .834 |
|  |  | Time | 0.972 | 0.000 | T0-T1: 0.00, *p* = .924  T1-T2: -0.01, *p* = .924  T0-T2: -0.01, *p* = .924 |
|  |  | Group × Time | 0.957 | 0.000 | Intervention:  T0-T1: 0.01, *p* = .968  T1-T2: -0.01, *p* = .968  T0-T2: -0.00, *p* = .968  Sham:  T0-T1: -0.01, *p* = .846  T1-T2: -0.01, *p* = .846;  T0-T2: -0.02, *p* = .846 |
|  | IL-6 | Group | 0.737 | 0.002 | T0: Intervention-Sham: -0.02, *p* = .573  T1: Intervention-Sham: 0.01, *p* = .711  T2: Intervention-Sham: -0.04, *p* = .148 |
|  |  | Time | **< .001** | 0.024 | T0-T1: 0.01, *p* = .474  T1-T2: -0.06, ***p* < .001**  T0-T2: -0.05, ***p* < .001** |
|  |  | Group × Time | 0.763 | 0.004 | Intervention:  T0-T1: -0.00, *p* = .933  T1-T2: -0.04, *p* = .099  T0-T2: -0.04, *p* = .099  Sham:  T0-T1: 0.03, *p* = .248  T1-T2: -0.09, ***p* < .001**  T0-T2: -0.07, ***p* = .004** |
|  | IL-1β | Group | 0.745 | 0.000 | T0: Intervention-Sham: 0.01, *p* = .774  T1: Intervention-Sham: 0.04, *p* = .242  T2: Intervention-Sham: -0.02, *p* = .537 |
|  |  | Time | **< .001** | 0.036 | T0-T1: 0.05, ***p* < .001**  T1-T2: -0.09, ***p* < .001**  T0-T2: -0.04, ***p* = .004** |
|  |  | Group × Time | 0.763 | 0.004 | Intervention:  T0-T1: 0.04, *p* = .090  T1-T2: -0.07, ***p* = .003**  T0-T2: -0.03, *p* = .124  Sham:  T0-T1: 0.07, ***p* = .003**  T1-T2: -0.13, ***p* < .001**  T0-T2: -0.06, ***p* = .007** |
|  | IL-10 | Group | 0.737 | 0.001 | T0: Intervention-Sham: -0.00, *p* = .949  T1: Intervention-Sham: 0.00, *p* = .980  T2: Intervention-Sham: -0.05, *p* = .330 |
|  |  | Time | 0.778 | 0.002 | T0-T1: 0.03, *p* = .523  T1-T2: -0.02, *p* = .523  T0-T2: 0.00, *p* = .879 |
|  |  | Group × Time | 0.804 | 0.002 | Intervention:  T0-T1: 0.02, *p* = .667  T1-T2: -0.00, *p* = .996  T0-T2: 0.02, *p* = .667  Sham:  T0-T1: 0.03, *p* = .530  T1-T2: -0.05, *p* = .485  T0-T2: -0.02, *p* = .530 |
|  | TNF-α | Group | 0.567 | 0.012 | T0: Intervention-Sham: -0.04, *p* = .200  T1: Intervention-Sham: -0.04, *p* = .282  T2: Intervention-Sham: -0.05, *p* = .118 |
|  |  | Time | **< .001** | 0.046 | T0-T1: -0.00, *p* = .991  T1-T2: 0.10, ***p* < .001**  T0-T2: 0.10, ***p* < .001** |
|  |  | Group × Time | 0.957 | 0.001 | Intervention:  T0-T1: -0.00, *p* = .903  T1-T2: 0.10, ***p* < .001**  T0-T2: 0.10, ***p* < .001**  Sham:  T0-T1: 0.00, *p* = .906  T1-T2: 0.09, ***p* < .001**  T0-T2: 0.09, ***p* < .001** |
| Anthropometrics | BMI | Group | 0.650 | 0.007 | T0: Intervention-Sham: -0.68, *p* = .371  T1: Intervention-Sham: -0.89, *p* = .259  T2: Intervention-Sham: -0.79, *p* = .327 |
|  |  | Time | **0.046** | 0.007 | T0-T1: 0.12, *p* = .710  T1-T2: 0.77, ***p* = .031**  T0-T2: 0.88, ***p* = .021** |
|  |  | Group × Time | 0.946 | 0.000 | Intervention:  T0-T1: 0.21, *p* = .619  T1-T2: 0.72, *p* = .173  T0-T2: 0.94, *p* = .117  Sham:  T0-T1: 0.01, *p* = .990  T1-T2: 0.82, *p* = .132  T0-T2: 0.82, *p* = .132 |
|  | SM | Group | 0.775 | 0.000 | T0: Intervention-Sham: -0.09, *p* = .869  T1: Intervention-Sham: -0.25, *p* = .649  T2: Intervention-Sham: -0.11, *p* = .835 |
|  |  | Time | 0.506 | 0.000 | T0-T1: 0.05, *p* = .639  T1-T2: 0.07, *p* = .639  T0-T2: 0.12, *p* = .639 |
|  |  | Group × Time | 0.946 | 0.000 | Intervention:  T0-T1: 0.12, *p* = .548  T1-T2: 0.01, *p* = .952  T0-T2: 0.13, *p* = .548  Sham:  T0-T1: -0.04, *p* = .778  T1-T2: 0.14, *p* = .717  T0-T2: 0.10, *p* = .717 |
|  | SMI | Group | 0.650 | 0.004 | T0: Intervention-Sham: -0.08, *p* = .583  T1: Intervention-Sham: -0.12, *p* = .394  T2: Intervention-Sham: -0.12, *p* = .408 |
|  |  | Time | 0.506 | 0.000 | T0-T1: 0.02, *p* = .579  T1-T2: 0.02, *p* = .579  T0-T2: 0.04, *p* = .579 |
|  |  | Group × Time | 0.946 | 0.000 | Intervention:  T0-T1: 0.04, *p* = .577  T1-T2: 0.02, *p* = .611  T0-T2: 0.06, *p* = .542  Sham:  T0-T1: -0.01, *p* = .907  T1-T2: 0.03, *p* = .907  T0-T2: 0.02, *p* = .907 |

***Note.*** *Panel A reports domain-level p values obtained by combining outcome-level p values within each domain using the Aggregated Cauchy Association Test (ACAT);* *ACAT combines multiple, potentially dependent p values into a single domain-level p value; domain-level p values were Benjamini–Hochberg (BH) adjusted across domains separately for each effect. N significant outcomes (FDR) indicate the number of outcomes within each domain reaching the pre-specified outcome-level FDR threshold (BH-adjusted p < .05) for the respective effect. Panel B reports all analyzed outcomes and all three model effects (Group, Time, and Group × Time). Baseline = T0; post-intervention = T1; 8-month follow-up = T2. Outcome-level p(FDR) values are BH-adjusted within each domain separately for each effect across outcomes. Contrast estimates are based on estimated marginal means (emmeans), with p values BH-adjusted within each contrast family. Semi-partial R² indicates the unique variance explained by the respective effect. For Group, Intervention–Sham comparisons are reported separately at T0, T1, and T2. For Time, T0–T1, T1–T2, and T0–T2 comparisons are reported as estimated marginal mean contrasts averaged over groups. For Group × Time, the same time comparisons are reported separately within the Intervention and Sham groups. Contrasts are reported for all outcomes for completeness. Statistical inference should primarily be based on the corresponding FDR-adjusted omnibus tests; contrasts associated with non-significant omnibus effects are descriptive only. Falls Efficacy Scale-International (FES-I) score; Falls Efficacy Scale-International Avoidance Behavior (FES-IAB); Updated Perceived Control over Falling Scale (UP-CoF); Geriatric Anxiety Scale (GAS); Perceived Stress Scale 10 (PSS-10); EURQOL Visual Analogue Scale (EQ-5D VAS); Short Physical Performance Battery (SPPB)*; *cortisol and amylase awakening response (CAR, AAR); area under the curve with respect to the ground (AUCg); Interleukin 6 (IL-6); Interleukin 10 (IL-10); Interleukin 1 beta (IL-1β); tumor necrosis factor alpha (TNF-α); C-reactive protein (CRP) body mass index (BMI); skeletal muscle mass (SM); skeletal muscle mass index (SMI).* *Intention-to-treat analyses were conducted using the same linear mixed-effects models as the primary analyses*
