# Supplementary material for: Reducing concerns about falling in older adults: evaluation of a multi-component intervention and the role of stress and inflammation markers - long-term effects of the FEARFALL randomized controlled trial
Source: Aging Clin Exp Res. 2026 Sep 19;38(1):195. doi: 10.1007/s40520-026-03515-z (PMC13597564; doi:10.1007/s40520-026-03515-z)
Supplement: Supplementary file 3 [file 40520_2026_3515_MOESM3_ESM.docx]

**Reducing Concerns about Falling in Older Adults: Effectiveness of a multi-component intervention and the role of stress and inflammation markers - Long-term effects of the FEARFALL randomized controlled trial**

Sabine Britting^1#^, Anja Müller^2#^, Robert Kob^1^, Ellen Freiberger^1^, Nicolas Rohleder^2^

^#^*shared first author*

^1^ Institute for Biomedicine of Aging, Department of Internal Medicine-Geriatrics, Friedrich-Alexander-Universität Erlangen-Nürnberg, Nuremberg, Germany

^2^ Chair of Health Psychology, Department of Psychology, Friedrich-Alexander-Universität Erlangen-Nürnberg, Erlangen, Germany

**Corresponding author**

Sabine Britting, Kobergerstr. 60, 90408 Nuremberg, Germany

Email: sabine.britting@fau.de

**ORCID**

Sabine Britting	0000-0001-8237-6105 23

Anja Müller	0009-0004-4101-9178 28

Robert Kob	0000-0002-0406-092X

Ellen Freiberger	0000-0002-6506-6276 26

Nicolas Rohleder	0000-0003-2602-517X 2

**Supplementary Information 3. Complete domain-level and outcome-level mixed-model results and BH-adjusted contrasts.**

**Panel A. Domain-level evidence.**

| **Domain** | **Effect** | **N outcomes** | **n significant outcomes (FDR)** | **ACAT *p* (raw)** | **ACAT *p* (adjusted)** |
| --- | --- | --- | --- | --- | --- |
| Psychological outcomes | Group | 7 | 0 | .917 | .917 |
|  | Time | 7 | 5 | **< .001** | **< .001** |
|  | Group × Time | 7 | 0 | .121 | .241 |
| Functional outcomes | Group | 10 | 0 | .832 | .917 |
|  | Time | 10 | 5 | **< .001** | **< .001** |
|  | Group × Time | 10 | 0 | .091 | .241 |
| Biomarkers (saliva + blood) | Group | 11 | 0 | .594 | .917 |
|  | Time | 11 | 5 | **< .001** | **< .001** |
|  | Group × Time | 11 | 0 | .915 | .950 |
| Anthropometrics | Group | 3 | 0 | .762 | .917 |
|  | Time | 3 | 0 | .068 | .068 |
|  | Group × Time | 3 | 0 | .950 | .950 |

**Panel B. Univariate outcome-level effects.**

| **Domain** | **Outcome** | **Effect** | ***p*(FDR)** | **Semi-partial *R^2^*** | **Contrasts (estimate, BH-adjusted *p*)** |
| --- | --- | --- | --- | --- | --- |
| Psychological outcomes | FES-I | Group | .517 | 0.005 | T0: Intervention-Sham: -0.28, *p* = .842 T1: Intervention-Sham: -2.10, *p* = .146 T2: Intervention-Sham: -1.15, *p* = .434 |
|  |  | Time | **< .001** | 0.060 | T0-T1: 4.48, *p* < .**001** T1-T2: -0.63, *p* = .418 T0-T2: 3.85, *p* < .**001** |
|  |  | Group × Time | .493 | 0.002 | Intervention: T0-T1: 5.34, *p* < .**001** T1-T2: -1.07, *p* = .310 T0-T2: 4.26, *p* < .**001** Sham: T0-T1: 3.51, *p* = .**004** T1-T2: -0.12, *p* = .914 T0-T2: 3.39, *p* = .**004** |
|  | FES-IAB | Group | .852 | 0.001 | T0: Intervention-Sham: 0.44, *p* = .700 T1: Intervention-Sham: -1.36, *p* = .245 T2: Intervention-Sham: -0.02, *p* = .984 |
|  |  | Time | **< .001** | 0.035 | T0-T1: 2.90, *p* < .**001** T1-T2: -0.78, *p* = .212 T0-T2: 2.12, *p* = .**001** |
|  |  | Group × Time | .493 | 0.003 | Intervention: T0-T1: 3.75, *p* < .**001** T1-T2: -1.40, *p* = .100 T0-T2: 2.35, *p* = .**009** Sham: T0-T1: 1.95, *p* = .**058** T1-T2: -0.07, *p* = .941 T0-T2: 1.88, *p* = .**058** |
|  | UP-CoF | Group | .223 | 0.020 | T0: Intervention-Sham: 0.68, *p* = .340 T1: Intervention-Sham: 1.89, *p* = .**007** T2: Intervention-Sham: 0.61, *p* = .388 |
|  |  | Time | **< .001** | 0.065 | T0-T1: -2.42, *p* < .**001** T1-T2: 0.74, *p* = .087 T0-T2: -1.68, *p* < .**001** |
|  |  | Group × Time | .493 | 0.006 | Intervention: T0-T1: -3.01, *p* < .**001** T1-T2: 1.34, *p* = .**023** T0-T2: -1.67, *p* = .**011** Sham: T0-T1: -1.80, *p* = .**009** T1-T2: 0.05, *p* = .930 T0-T2: -1.74, *p* = .**009** |
|  | GAS | Group | .517 | 0.008 | T0: Intervention-Sham: -2.49, *p* = .064 T1: Intervention-Sham: -1.88, *p* = .169 T2: Intervention-Sham: 0.56, *p* = .683 |
|  |  | Time | **< .001** | 0.016 | T0-T1: 2.02, *p* = .**002** T1-T2: -0.14, *p* = .819 T0-T2: 1.88, *p* = .**003** |
|  |  | Group × Time | .183 | 0.007 | Intervention: T0-T1: 1.73, *p* = .095 T1-T2: -1.28, *p* = .179 T0-T2: 0.45, *p* = .581 Sham: T0-T1: 2.34, *p* = .**009** T1-T2: 1.16, *p* = .184 T0-T2: 3.50, *p* < .**001** |
|  | GDS | Group | .396 | 0.015 | T0: Intervention-Sham: -0.72, *p* = .**051** T1: Intervention-Sham: -0.47, *p* = .203 T2: Intervention-Sham: -0.26, *p* = .486 |
|  |  | Time | .050 | 0.008 | T0-T1: 0.43, *p* = .**045** T1-T2: -0.18, *p* = .308 T0-T2: 0.25, *p* = .253 |
|  |  | Group × Time | .493 | 0.002 | Intervention: T0-T1: 0.32, *p* = .384 T1-T2: -0.28, *p* = .384 T0-T2: 0.04, *p* = .883 Sham: T0-T1: 0.56, *p* = .090 T1-T2: -0.07, *p* = .793 T0-T2: 0.49, *p* = .095 |
|  | PSS | Group | .590 | 0.004 | T0: Intervention-Sham: -0.52, *p* = .655 T1: Intervention-Sham: -2.08, *p* = .080 T2: Intervention-Sham: 0.19, *p* = .876 |
|  |  | Time | **.007** | 0.012 | T0-T1: 1.51, *p* = .**010** T1-T2: -0.06, *p* = .914 T0-T2: 1.46, *p* = .**010** |
|  |  | Group × Time | .320 | 0.005 | Intervention: T0-T1: 2.25, *p* = .**006** T1-T2: -1.12, *p* = .128 T0-T2: 1.13, *p* = .128 Sham: T0-T1: 0.69, *p* = .365 T1-T2: 1.15, *p* = .214 T0-T2: 1.84, *p* = .055 |
|  | Lifequal  (EQ-5D VAS) | Group | .992 | 0.000 | T0: Intervention-Sham: 0.46, *p* = .887 T1: Intervention-Sham: 1.49, *p* = .651 T2: Intervention-Sham: -2.02, *p* = .544 |
|  |  | Time | .056 | 0.007 | T0-T1: -3.37, *p* = .071 T1-T2: 0.74, *p* = .628 T0-T2: -2.63, *p* = .122 |
|  |  | Group × Time | .493 | 0.002 | Intervention: T0-T1: -3.85, *p* = .179 T1-T2: 2.38, *p* = .382 T0-T2: -1.47, *p* = .478 Sham: T0-T1: -2.82, *p* = .288 T1-T2: -1.13, *p* = .611 T0-T2: -3.95, *p* = .220 |
| Functional outcomes | Handgrip strength (kg) | Group | .871 | 0.002 | T0: Intervention-Sham: 0.74, *p* = .482 T1: Intervention-Sham: 1.38, *p* = .192 T2: Intervention-Sham: 0.21, *p* = .846 |
|  |  | Time | **< .001** | 0.015 | T0-T1: -0.76, *p* = .**040** T1-T2: -1.67, *p* < .**001** T0-T2: -2.43, *p* < .**001** |
|  |  | Group × Time | .471 | 0.001 | Intervention: T0-T1: -1.07, *p* = .**034** T1-T2: -1.12, *p* = .**034** T0-T2: -2.19, *p* < .**001** Sham: T0-T1: -0.43, *p* = .432 T1-T2: -2.29, *p* < .**001** T0-T2: -2.72, *p* < .**001** |
|  | PA-Level | Group | .971 | 0.000 | T0: Intervention-Sham: 0.03, *p* = .792 T1: Intervention-Sham: 0.00, *p* = .992 T2: Intervention-Sham: -0.04, *p* = .725 |
|  |  | Time | .066 | 0.008 | T0-T1: -0.09, *p* = .158 T1-T2: 0.15, *p* = .036 T0-T2: 0.05, *p* = .344 |
|  |  | Group × Time | .801 | 0.001 | Intervention: T0-T1: -0.08, *p* = .320 T1-T2: 0.17, *p* = .108 T0-T2: 0.09, *p* = .320 Sham: T0-T1: -0.11, *p* = .289 T1-T2: 0.12, *p* = .289 T0-T2: 0.01, *p* = .869 |
|  | SPPB  (total Score) | Group | .971 | 0.000 | T0: Intervention-Sham: 0.18, *p* = .673 T1: Intervention-Sham: -0.55, *p* = .212 T2: Intervention-Sham: 0.28, *p* = .536 |
|  |  | Time | **.046** | 0.004 | T0-T1: -0.32, *p* = .069 T1-T2: -0.10, *p* = .550 T0-T2: -0.47, *p* = .**034** |
|  |  | Group × Time | .174 | 0.005 | Intervention: T0-T1: 0.01, *p* = .910 T1-T2: -0.41, *p* = .059 T0-T2: -0.45, *p* = .059 Sham: T0-T1: -0.71, *p* = .**006** T1-T2: 0.35, *p* = .141 T0-T2: -0.35, *p* = .141 |
|  | SPPB (balance) | Group | .971 | 0.000 | T0: Intervention-Sham: 0.25, *p* = .362 T1: Intervention-Sham: -0.39, *p* = .188 T2: Intervention-Sham: 0.06, *p* = .890 |
|  |  | Time | .139 | 0.011 | T0-T1: 0.06, *p* = .682 T1-T2: -0.48, *p* = .081 T0-T2: -0.42, *p* = .081 |
|  |  | Group × Time | .247 | 0.007 | Intervention: T0-T1: 0.36, *p* = .125 T1-T2: -0.67, *p* = .**043** T0-T2: -0.31, *p* = .241 Sham: T0-T1: -0.28, *p* = .307 T1-T2: -0.21, *p* = .552 T0-T2: -0.50, *p* = .307 |
|  | SPPB (4m [score 1-4]) | Group | .871 | 0.012 | T0: Intervention-Sham: 0.27, *p* = .032 T1: Intervention-Sham: 0.06, *p* = .622 T2: Intervention-Sham: 0.12, *p* = .378 |
|  |  | Time | .**046** | 0.004 | T0-T1: -0.12, *p* = .**036** T1-T2: 0.04, *p* = .415 T0-T2: -0.08, *p* = .150 |
|  |  | Group × Time | .247 | 0.003 | Intervention: T0-T1: -0.03, *p* = .890 T1-T2: 0.02, *p* = .890 T0-T2: -0.01, *p* = .890 Sham: T0-T1: -0.24, *p* = .003 T1-T2: 0.07, *p* = .363 T0-T2: -0.17, *p* = .**036** |
|  | SPPB (4m speed [m/sec]) | Group | .871 | 0.005 | T0: Intervention-Sham: 0.04, *p* = .198 T1: Intervention-Sham: 0.03, *p* = .409 T2: Intervention-Sham: 0.01, *p* = .767 |
|  |  | Time | **.004** | 0.008 | T0-T1: -0.05, *p* < .**001** T1-T2: 0.02, *p* = .070 T0-T2: -0.02, *p* = .070 |
|  |  | Group × Time | .532 | 0.001 | Intervention: T0-T1: -0.04, *p* = .062 T1-T2: 0.03, *p* = .109 T0-T2: -0.01, *p* = .620 Sham: T0-T1: -0.06, *p* = .008 T1-T2: 0.01, *p* = .501 T0-T2: -0.04, *p* = .**042** |
|  | SPPB (chair rise [score 1-4] | Group | .871 | 0.004 | T0: Intervention-Sham: -0.13, *p* = .573 T1: Intervention-Sham: -0.31, *p* = .204 T2: Intervention-Sham: -0.04, *p* = .860 |
|  |  | Time | .084 | 0.005 | T0-T1: -0.22, *p* = .084 T1-T2: 0.14, *p* = .267 T0-T2: -0.08, *p* = .426 |
|  |  | Group × Time | .532 | 0.002 | Intervention: T0-T1: -0.14, *p* = .586 T1-T2: 0.02, *p* = .873 T0-T2: -0.12, *p* = .586 Sham: T0-T1: -0.32, *p* = .098 T1-T2: 0.29, *p* = .100 T0-T2: -0.03, *p* = .844 |
|  | SPPB (chair rise [sec]) | Group | .896 | 0.001 | T0: Intervention-Sham: -1.73, *p* = .229 T1: Intervention-Sham: 0.86, *p* = .569 T2: Intervention-Sham: 2.47, *p* = .122 |
|  |  | Time | .082 | 0.018 | T0-T1: -0.44, *p* = .676 T1-T2: -2.11, *p* = .083 T0-T2: -2.55, *p* = .056 |
|  |  | Group × Time | .263 | 0.012 | Intervention: T0-T1: -1.67, *p* = .243 T1-T2: -2.84, *p* = .085 T0-T2: -4.51, *p* = .**007** Sham: T0-T1: 0.92, *p* = .815 T1-T2: -1.23, *p* = .815 T0-T2: -0.31, *p* = .846 |
|  | Steps (daily average) | Group | .871 | 0.008 | T0: Intervention-Sham: 175.67, *p* = .742 T1: Intervention-Sham: 434.04, *p* = .423 T2: Intervention-Sham: 1114.88, *p* = .**042** |
|  |  | Time | **.044** | 0.006 | T0-T1: 377.38, *p* = .082 T1-T2: 148.67, *p* = .456 T0-T2: 526.04, *p* = .**026** |
|  |  | Group × Time | .247 | 0.004 | Intervention: T0-T1: 255.09, *p* = .719 T1-T2: -158.78, *p* = .719 T0-T2: 96.31, *p* = .719 Sham: T0-T1: 513.46, *p* = .077 T1-T2: 522.06, *p* = .077 T0-T2: 1035.52, *p* = .**001** |
|  | Falls | Group | .971 | 0.001 | T0: Intervention-Sham: -0.01, *p* = .866 T1: Intervention-Sham: 0.01, *p* = .897 T2: Intervention-Sham: -0.04, *p* = .419 |
|  |  | Time | .599 | 0.001 | T0-T1: 0.01, *p* = .688 T1-T2: 0.01, *p* = .688 T0-T2: 0.02, *p* = .688 |
|  |  | Group × Time | .595 | 0.001 | Intervention: T0-T1: 0.00, *p* = .958 T1-T2: 0.04, *p* = .333 T0-T2: 0.04, *p* = .333 Sham: T0-T1: 0.02, *p* = .863 T1-T2: -0.01, *p* = .863 T0-T2: 0.01, *p* = .863 |
| Biomarkers | CAR | Group | .910 | 0.005 | T0: Intervention-Sham: -0.01, *p* = .820 T1: Intervention-Sham: 0.01, *p* = .786 T2: Intervention-Sham: 0.10, *p* = .**031** |
|  |  | Time | .728 | 0.002 | T0-T1: -0.02, *p* = .750 T1-T2: 0.02, *p* = .750 T0-T2: -0.00, *p* = .895 |
|  |  | Group × Time | .983 | 0.012 | Intervention: T0-T1: -0.03, *p* = .532 T1-T2: -0.02, *p* = .548 T0-T2: -0.05, *p* = .428 Sham: T0-T1: -0.01, *p* = .797 T1-T2: 0.07, *p* = .260 T0-T2: 0.05, *p* = .260 |
|  | Cortisol Slope | Group | .910 | 0.002 | T0: Intervention-Sham: 0.00, *p* = .678 T1: Intervention-Sham: -0.00, *p* = .225 T2: Intervention-Sham: -0.00, *p* = .587 |
|  |  | Time | .993 | 0.000 | T0-T1: 0.00, *p* = .974 T1-T2: -0.00, *p* = .974 T0-T2: 0.00, *p* = .974 |
|  |  | Group × Time | .983 | 0.005 | Intervention: T0-T1: 0.00, *p* = .731 T1-T2: -0.00, *p* = .731 T0-T2: 0.00, *p* = .731 Sham: T0-T1: -0.00, *p* = .685 T1-T2: 0.00, *p* = .685 T0-T2: -0.00, *p* = .685 |
|  | Cortisol (AUCg) | Group | .910 | 0.000 | T0: Intervention-Sham: -0.23, *p* = .608 T1: Intervention-Sham: 0.08, *p* = .867 T2: Intervention-Sham: 0.41, *p* = .392 |
|  |  | Time | **< .001** | 0.042 | T0-T1: -0.58, *p* = .**021** T1-T2: 1.24, *p* < .**001** T0-T2: 0.66, *p* = .**017** |
|  |  | Group × Time | .983 | 0.003 | Intervention: T0-T1: -0.71, *p* = .**047** T1-T2: 1.08, *p* = .**010** T0-T2: 0.37, *p* = .299 Sham: T0-T1: -0.40, *p* = .293 T1-T2: 1.41, *p* = .**001** T0-T2: 1.01, *p* = .**014** |
|  | AAR | Group | .910 | 0.006 | T0: Intervention-Sham: -0.13, *p* = .794 T1: Intervention-Sham: -0.35, *p* = .515 T2: Intervention-Sham: -0.81, *p* = .159 |
|  |  | Time | .718 | 0.004 | T0-T1: 0.14, *p* = .679 T1-T2: -0.40, *p* = .679 T0-T2: -0.26, *p* = .679 |
|  |  | Group × Time | .983 | 0.003 | Intervention: T0-T1: 0.23, *p* = .915 T1-T2: -0.18, *p* = .915 T0-T2: 0.05, *p* = .915 Sham: T0-T1: 0.02, *p* = .975 T1-T2: -0.65, *p* = .358 T0-T2: -0.63, *p* = .358 |
|  | Amylase Slope | Group | .910 | 0.000 | T0: Intervention-Sham: 0.00, *p* = .984 T1: Intervention-Sham: 0.01, *p* = .857 T2: Intervention-Sham: 0.00, *p* = .969 |
|  |  | Time | .718 | 0.003 | T0-T1: 0.02, *p* = .607 T1-T2: -0.03, *p* = .607 T0-T2: -0.01, *p* = .769 |
|  |  | Group × Time | .989 | 0.000 | Intervention: T0-T1: 0.02, *p* = .824 T1-T2: -0.02, *p* = .824 T0-T2: -0.01, *p* = .824 Sham: T0-T1: 0.02, *p* = .777 T1-T2: -0.03, *p* = .777 T0-T2: -0.01, *p* = .856 |
|  | Amylase (AUCg) | Group | .910 | 0.003 | T0: Intervention-Sham: -3.77, *p* = .674 T1: Intervention-Sham: -8.13, *p* = .381 T2: Intervention-Sham: -3.31, *p* = .729 |
|  |  | Time | **.007** | 0.013 | T0-T1: 3.09, *p* = .386 T1-T2: -13.16, *p* = .**003** T0-T2: -10.07, *p* = .**013** |
|  |  | Group × Time | .989 | 0.000 | Intervention: T0-T1: 4.90, *p* = .296 T1-T2: -15.23, *p* = .**012** T0-T2: -10.34, *p* = .068 Sham: T0-T1: 0.54, *p* = .923 T1-T2: -10.42, *p* = .131 T0-T2: -9.88, *p* = .131 |
|  | CRP | Group | .910 | 0.003 | T0: Intervention-Sham: 0.04, *p* = .412 T1: Intervention-Sham: 0.01, *p* = .850 T2: Intervention-Sham: 0.03, *p* = .552 |
|  |  | Time | .728 | 0.002 | T0-T1: 0.00, *p* = .937 T1-T2: -0.02, *p* = .615 T0-T2: -0.02, *p* = .615 |
|  |  | Group × Time | .989 | 0.001 | Intervention: T0-T1: 0.01, *p* = .643 T1-T2: -0.03, *p* = .643 T0-T2: -0.01, *p* = .643 Sham: T0-T1: -0.01, *p* = .742 T1-T2: -0.01, *p* = .742 T0-T2: -0.02, *p* = .742 |
|  | IL-6 | Group | .910 | 0.000 | T0: Intervention-Sham: -0.00, *p* = .982 T1: Intervention-Sham: 0.01, *p* = .668 T2: Intervention-Sham: -0.03, *p* = .326 |
|  |  | Time | **< .001** | 0.025 | T0-T1: 0.01, *p* = .731 T1-T2: -0.06, *p* = .**001** T0-T2: -0.06, *p* = .**002** |
|  |  | Group × Time | .983 | 0.003 | Intervention: T0-T1: -0.00, *p* = .961 T1-T2: -0.04, *p* = .119 T0-T2: -0.04, *p* = .119 Sham: T0-T1: 0.01, *p* = .575 T1-T2: -0.09, *p* = .**002** T0-T2: -0.07, *p* = .**005** |
|  | IL-1β | Group | .910 | 0.001 | T0: Intervention-Sham: 0.02, *p* = .614 T1: Intervention-Sham: 0.04, *p* = .297 T2: Intervention-Sham: -0.01, *p* = .834 |
|  |  | Time | **< .001** | 0.038 | T0-T1: 0.05, *p* = .**002** T1-T2: -0.10, *p* < .**001** T0-T2: -0.05, *p* = .**004** |
|  |  | Group × Time | .983 | 0.003 | Intervention: T0-T1: 0.04, *p* = .087 T1-T2: -0.08, *p* = .**002** T0-T2: -0.04, *p* = .099 Sham: T0-T1: 0.06, *p* = .**010** T1-T2: -0.12, *p* < .**001** T0-T2: -0.06, *p* = .**011** |
|  | IL-10 | Group | .910 | 0.000 | T0: Intervention-Sham: 0.01, *p* = .828 T1: Intervention-Sham: 0.00, *p* = .959 T2: Intervention-Sham: -0.04, *p* = .506 |
|  |  | Time | .557 | 0.003 | T0-T1: 0.04, *p* = .422 T1-T2: -0.03, *p* = .484 T0-T2: 0.01, *p* = .670 |
|  |  | Group × Time | .983 | 0.002 | Intervention: T0-T1: 0.04, *p* = .510 T1-T2: -0.01, *p* = .821 T0-T2: 0.03, *p* = .510 Sham: T0-T1: 0.03, *p* = .555 T1-T2: -0.05, *p* = .555 T0-T2: -0.02, *p* = .670 |
|  | TNF-α | Group | .910 | 0.018 | T0: Intervention-Sham: -0.06, *p* = .119 T1: Intervention-Sham: -0.05, *p* = .166 T2: Intervention-Sham: -0.05, *p* = .186 |
|  |  | Time | **< .001** | 0.040 | T0-T1: -0.01, *p* = .701 T1-T2: 0.09, *p* < .**001** T0-T2: 0.09, *p* < .**001** |
|  |  | Group × Time | .989 | 0.000 | Intervention: T0-T1: -0.01, *p* = .692 T1-T2: 0.09, *p* < .**001** T0-T2: 0.08, *p* < .**001** Sham: T0-T1: -0.00, *p* = .886 T1-T2: 0.09, *p* < .**001** T0-T2: 0.09, *p* < .**001** |
| Anthropometrics | BMI | Group | .728 | 0.005 | T0: Intervention-Sham: -0.61, *p* = .474 T1: Intervention-Sham: -0.74, *p* = .387 T2: Intervention-Sham: -0.67, *p* = .447 |
|  |  | Time | .079 | 0.007 | T0-T1: 0.05, *p* = .898 T1-T2: 0.85, *p* = .**034** T0-T2: 0.90, *p* = .**034** |
|  |  | Group × Time | .982 | 0.000 | Intervention: T0-T1: 0.11, *p* = .823 T1-T2: 0.81, *p* = .176 T0-T2: 0.93, *p* = .176 Sham: T0-T1: -0.02, *p* = .962 T1-T2: 0.89, *p* = .159 T0-T2: 0.86, *p* = .159 |
|  | SM | Group | .917 | 0.000 | T0: Intervention-Sham: 0.13, *p* = .833 T1: Intervention-Sham: -0.03, *p* = .967 T2: Intervention-Sham: 0.09, *p* = .894 |
|  |  | Time | .309 | 0.000 | T0-T1: 0.07, *p* = .517 T1-T2: 0.10, *p* = .517 T0-T2: 0.17, *p* = .380 |
|  |  | Group × Time | .982 | 0.000 | Intervention: T0-T1: 0.15, *p* = .473 T1-T2: 0.04, *p* = .774 T0-T2: 0.19, *p* = .473 Sham: T0-T1: -0.02, *p* = .912 T1-T2: 0.16, *p* = .554 T0-T2: 0.14, *p* = .554 |
|  | SMI | Group | .728 | 0.003 | T0: Intervention-Sham: -0.08, *p* = .640 T1: Intervention-Sham: -0.12, *p* = .471 T2: Intervention-Sham: -0.13, *p* = .423 |
|  |  | Time | .309 | 0.001 | T0-T1: 0.03, *p* = .440 T1-T2: 0.03, *p* = .440 T0-T2: 0.06, *p* = .265 |
|  |  | Group × Time | .982 | 0.000 | Intervention: T0-T1: 0.05, *p* = .411 T1-T2: 0.04, *p* = .411 T0-T2: 0.09, *p* = .238 Sham: T0-T1: 0.01, *p* = .917 T1-T2: 0.03, *p* = .896 T0-T2: 0.03, *p* = .896 |

***Note.*** *Panel A reports domain-level p values obtained by combining outcome-level p values within each domain using the Aggregated Cauchy Association Test (ACAT); ACAT combines multiple, potentially dependent p values into a single domain-level p value; domain-level p values were Benjamini–Hochberg (BH) adjusted across domains separately for each effect. N significant outcomes (FDR) indicate the number of outcomes within each domain reaching the pre-specified outcome-level FDR threshold (BH-adjusted p < .05) for the respective effect. Panel B reports all analyzed outcomes and all three model effects (Group, Time, and Group × Time). Baseline = T0; post-intervention = T1; 8-month follow-up = T2. Outcome-level p(FDR) values are BH-adjusted within each domain separately for each effect across outcomes. Contrast estimates are based on estimated marginal means (emmeans), with p values BH-adjusted within each contrast family. Semi-partial R² indicates the unique variance explained by the respective effect. For Group, Intervention–Sham comparisons are reported separately at T0, T1, and T2. For Time, T0–T1, T1–T2, and T0–T2 comparisons are reported as estimated marginal mean contrasts averaged over groups. For Group × Time, the same time comparisons are reported separately within the Intervention and Sham groups. Contrasts are reported for all outcomes for completeness. Statistical inference should primarily be based on the corresponding FDR-adjusted omnibus tests; contrasts associated with non-significant omnibus effects are descriptive only.. Falls Efficacy Scale-International (FES-I) score; Falls Efficacy Scale-International Avoidance Behavior (FES-IAB); Updated Perceived Control over Falling Scale (UP-CoF); Geriatric Anxiety Scale (GAS); Perceived Stress Scale 10 (PSS-10); EURQOL Visual Analogue Scale (EQ-5D VAS); Short Physical Performance Battery (SPPB)*; *cortisol and amylase awakening response (CAR, AAR); area under the curve with respect to the ground (AUCg); Interleukin 6 (IL-6); Interleukin 10 (IL-10); Interleukin 1 beta (IL-1β); tumor necrosis factor alpha (TNF-α); C-reactive protein (CRP) body mass index (BMI); skeletal muscle mass (SM); skeletal muscle mass index (SMI).*
