# Supplementary material for: Reducing concerns about falling in older adults: evaluation of a multi-component intervention and the role of stress and inflammation markers - long-term effects of the FEARFALL randomized controlled trial
Source: Aging Clin Exp Res. 2026 Sep 19;38(1):195. doi: 10.1007/s40520-026-03515-z (PMC13597564; doi:10.1007/s40520-026-03515-z)
Supplement: Supplementary file 4 [file 40520_2026_3515_MOESM4_ESM.docx]

**Reducing Concerns about Falling in Older Adults: Effectiveness of a multi-component intervention and the role of stress and inflammation markers - Long-term effects of the FEARFALL randomized controlled trial**

Sabine Britting^1#^, Anja Müller^2#^, Robert Kob^1^, Ellen Freiberger^1^, Nicolas Rohleder^2^

^#^*shared first author*

^1^ Institute for Biomedicine of Aging, Department of Internal Medicine-Geriatrics, Friedrich-Alexander-Universität Erlangen-Nürnberg, Nuremberg, Germany

^2^ Chair of Health Psychology, Department of Psychology, Friedrich-Alexander-Universität Erlangen-Nürnberg, Erlangen, Germany

**Corresponding author**

Sabine Britting, Kobergerstr. 60, 90408 Nuremberg, Germany

Email: sabine.britting@fau.de

**ORCID**

Sabine Britting	0000-0001-8237-6105 23

Anja Müller	0009-0004-4101-9178 28

Robert Kob	0000-0002-0406-092X

Ellen Freiberger	0000-0002-6506-6276 26

Nicolas Rohleder	0000-0003-2602-517X 2

**Supplementary Information 2: Flow chart Study population**

***Note*.** IG = intervention group; SCG = sham control group; T1 = post-intervention assessment; T2 = 8-month follow-up assessment. Program completion was defined as attendance at ≥ 75% of scheduled sessions and constituted the basis for the per-protocol sample.
